# Significance of hydrogen bonding and other noncovalent interactions in determining octahedral tilting in the CH_3_NH_3_PbI_3_ hybrid organic-inorganic halide perovskite solar cell semiconductor

**DOI:** 10.1038/s41598-018-36218-1

**Published:** 2019-01-10

**Authors:** Pradeep R. Varadwaj, Arpita Varadwaj, Helder M. Marques, Koichi Yamashita

**Affiliations:** 1 Department of Chemical System Engineering, School of Engineering, The University of Tokyo 7-3-1, Hongo, Bunkyo-ku 113-8656 Japan; 2 CREST-JST, 7 Gobancho, Chiyoda-ku, Tokyo 102-0076 Japan; 3 The National Institute of Advanced Industrial Science and Technology (AIST), Tsukuba, Ibaraki 305-8560 Japan; 4 Molecular Sciences Institute, School of Chemistry, University of the Witwatersrand, Johannesburg, 2050 South Africa

## Abstract

The CH_3_NH_3_PbI_3_ (methylammonium lead triiodide) perovskite semiconductor system has been viewed as a blockbuster research material during the last five years. Because of its complicated architecture, several of its technological, physical and geometrical issues have been examined many times. Yet this has not assisted in overcoming a number of problems in the field nor in enabling the material to be marketed. For instance, these studies have not clarified the nature and type of hydrogen bonding and other noncovalent interactions involved; the origin of hysteresis; the actual role of the methylammonium cation; the nature of polarity associated with the tetragonal geometry; the unusual origin of various frontier orbital contributions to the conduction band minimum; the underlying phenomena of spin-orbit coupling that causes significant bandgap reduction; and the nature of direct-to-indirect bandgap transition features. Arising from many recent reports, it is now a common belief that the I···H–N interaction formed between the inorganic framework and the ammonium group of CH_3_NH_3_^+^ is the only hydrogen bonded interaction responsible for all temperature-dependent geometrical polymorphs of the system, including the most stable one that persists at low-temperatures, and the significance of all other noncovalent interactions has been overlooked. This study focussed only on the low temperature orthorhombic polymorph of CH_3_NH_3_PbI_3_ and CD_3_ND_3_PbI_3_, where D refers deuterium. Together with QTAIM, DORI and RDG based charge density analyses, the results of density functional theory calculations with PBE with and without van der Waals corrections demonstrate that the prevailing view of hydrogen bonding in CH_3_NH_3_PbI_3_ is misleading as it does not alone determine the *a*^−^*b*^+^*a*^−^ tilting pattern of the PbI_6_^4−^ octahedra. This study suggests that it is not only the I···H/D–N, but also the I···H/D–C hydrogen/deuterium bonding and other noncovalent interactions (*viz*. tetrel-, pnictogen- and lump-hole bonding interactions) that are ubiquitous in the orthorhombic CH_3_NH_3_PbI_3_/CD_3_ND_3_PbI_3_ perovskite geometry. Their interplay determines the overall geometry of the polymorph, and are therefore responsible in part for the emergence of the functional optical properties of this material. This study also suggests that these interactions should not be regarded as the sole determinants of octahedral tilting since lattice dynamics is known to play a critical role as well, a common feature in many inorganic perovskites both in the presence and the absence of the encaged cation, as in CsPbI_3_/WO_3_ perovskites, for example.

## Introduction

Together with the nature of the atomic constituents, intra- and/or intermolecular noncovalent interactions (NCIs) play a crucial role in regulating ordering between and packing of the molecular domains, and hence are the determinants of the geometry of a crystal and its properties^1–6^. The studies of the structure of millions of crystals lodged in various structural databases^7^ have utilized the “less than the sum of the van der Waals radii (vdW)” as a principal criterion to identify and characterize intermolecular bonding interactions^8–11^, although this criterion fails on many occasions especially when a variety of such interactions are weak and of the vdW type^12–16^. Clearly, the nature of NCIs in similar systems needs to be carefully elucidated to avoid arriving at misleading conclusions and an incorrect interpretation of theoretically simulated and experimentally observed data.

The CH_3_NH_3_PbI_3_ (methylammonium lead triiodide, MAPbI_3_) semiconductor is one of such crystal systems wherein NCIs play an important role. This interesting system has been extensively investigated in the last couple of years, owing to its extraordinary efficiency as a solar energy converter; yet its physical properties have yet to be fully delineated^17–20^. Structural elements^21^, including the tilting of the PbI_6_^4−^ octahedra of the inorganic core^22–24^, play an important role in dictating the function of the material, and is known to be driven by noncovalent interactions. The extent of tilting of the MY_6_^4−^ octahedra in BMY_3_ halide perovskites (B = monovalent organic or inorganic cation, M = divalent metal, and Y = Cl, Br, I and their mixed derivatives) is usually quantified in terms of the deviations of the M—Y—M bond angles from 180° along the three crystallographic directions^21–26^. The origin of such tilting of the octahedra in inorganic and organic-inorganic perovskites has also shown to be a consequence of a (rotational) lattice disorder^22,27,28^, which is associated with order-disorder dynamics^29,30^. As has been pointed out elsewhere^20,22,27,28^, the dynamics of the local environment of perovskites directed in part by hydrogen bonding (an NCI) remains unclear; accurate simulations of lattice dynamics are critically needed to shed some light on how these impact the electronic properties of the material.

Both in- and anti-phase tilting of the PbI_6_^4−^ octahedra have been crystallographically observed in o-CD_3_ND_3_PbI_3_ and o-CH_3_NH_3_PbI_3_ perovskites^31,32^ (see below), and confirmed theoretically^24–26,32^ for the orthorhombic polymorph of CH_3_NH_3_PbI_3_ perovskite. The tilting is prominent along the *a* and *b* mutually perpendicular crystallographic axes described by *a*^*−*^*b*^+^*a*^*−*^ in Glazer notation^33^. The same tilting feature is also evident of all-inorganic perovskites, such as CsPbI_3_^34,35^, for example.

MAPbI_3_ exists in three main temperature phases (orthorhombic, *T* < 165 K; tetragonal, 165 < *T* < 327 K; and cubic, *T* > 327 K)^4,18,19,25,26,31,32,36^. Several studies have endeavored to shed light on geometrical aspects of the three phases of the system that are driven by different modes of hydrogen bonding. The way hydrogen bonding between the guest CH_3_NH_3_^+^ (MA) cation and the host lead iodide inorganic lattice affects the tilting of the PbI_6_^4−^ octahedra in the equilibrium geometry of o-CH_3_NH_3_PbI_3_^24–26^ has been demonstrated. While doing so, the presence, and importance, of other noncovalent interactions appear to have gone unnoticed, or unjustifiably underestimated. It was concluded that I···H–N hydrogen bonding interactions are mainly responsible for controlling the relative orientations of MA and the host lattice, and hence, driving the in-phase and out-of-phase rotations of the PbI_6_^4−^ octahedra. An effort was then made to correlate the bond distances associated with these interactions with the rotations of the octahedra in the perovskite lattice, an attempt, which, as we shall discuss below, is misleading. A similar attempt was made by others to answer questions such as: Does the inorganic framework deform on its own and the MA cation then accommodates itself in a particular orientation in the deformed cage? Or does the MA cation force the inorganic framework into a particular deformation^37^? The present investigation, however, suggests that these studies have overlooked the importance of I···H–C hydrogen bonding and several other noncovalent interactions that are inherent in o-CH_3_NH_3_PbI_3_, similar to effects that we^3^ and others^17^ have observed in another member of the same perovskite family, CH_3_NH_3_PbBr_3_. An exploration of the nature of noncovalent interactions in o-CH_3_NH_3_PbI_3_ is thus one of the core objectives of this investigation.

A distance criterion (H···I < 3 Å) appears to have been used to determine whether or not a hydrogen bonding interaction is present in o-CH_3_NH_3_PbI_3_^25^. However, what other criteria were used to identify and characterize such interactions in the crystallographic studies is unclear. For instance, Weller and coworkers^31^ performed a neutron diffraction study on o-CH_3_NH_3_PbI_3_ and reported some intermolecular distances between N and H atoms; they then suggested possible hydrogen bonds between them. However, what specific signatures (other than pair distribution function analysis) were used to claim that only I···H–N, but not I···H–C, interactions are feasible in the geometry of the system remain controversial. A similar view was presented in a study reported by Ren and coworkers^36^. Baikie and others *et al*.^38^ have also presented neutron diffraction data and their ^1^H MAS NMR spectra showed two clear peaks with equal populations corresponding to the –CH_3_ and –NH_3_ environments in the high temperature phase, yet the authors have observed the –CH_3_ but the not the –NH_3_ protons to have longer relaxation times just before the phase transition and the reason for this behavior was unclear; the authors attributed this to the nitrogen atom which is heavier than the carbon such that the –CH_3_ end of the ion is more likely to not to interact with the changing sites of the cage. Since then, many other studies have relied on these findings to claim the importance of I···H–N hydrogen bonding in the o-CH_3_NH_3_PbI_3_ system^31^, as well as the I···D–N deuterium bonding in the deuterated systems^32^, both experimentally^31,32,36^ and theoretically^31,32,36,39–52^. The other members of the same perovskite family, *viz*., o-CH_3_NH_3_PbCl_3_ and o-CH_3_NH_3_PbBr_3_, also adopt an orthorhombic polymorphic structure, meaning that hydrogen bonding is equally important for the overall structural stability and for the evolution of the material properties of these systems. To claim that the strength of hydrogen bonds between these three systems in the series is nearly equivalent^53^, in our view, is incorrect since the strength of hydrogen bonding formed by the covalently or metal-coordinated Cl, Br and I atoms in molecular and solid state systems with a given type of hydrogen bond donor should follow the general trend Cl > Br > I, which is true provided secondary interactions do not significantly hamper the status of the primary interactions. This trend is intuitively reasonable since the size of the halogen correlates inversely with its electronegativity and the strength of its hydrogen bonding interactions.

While it is relatively straightforward to identify hydrogen bonding interactions in systems such as o-CH_3_NH_3_PbI_3_, it seems to us that sufficient care has not been made to date to make use of the appropriate criteria and characteristic features that IUPAC recommended for the identification and characterization of such important geometrical features^54^. This is analogous to studies that have misleadingly referred to *hybrid organic-inorganic halide perovskites* as *organometallic perovskites*^55–58^, yet the notion continues in other studies to date. We^18^ and later by others^59^, argue that these compounds do not contain a metal-carbon framework; thus referring to them as organometallic compounds violates the underlying definition of the term “*organometallic*”, as put forwarded by IUPAC^60^. As said, appropriate characterization of intermolecular interactions in o-CH_3_NH_3_PbI_3_ is an essential step forward that might be used to assist to clarify the many misleading conclusions that have been advanced in this area. Also, elucidation of these fundamentally important interactions might shed some light on how these determine the overall shape of the material, contributing to tilting, and enabling it to display extraordinary optical and mechanical characteristics^61^ and eventually, causing it to become functional for possible applications in future device technology.

In this paper, using the established literature on noncovalent interactions and the results (geometrical, electronic and topological properties) of Density Functional Theory (DFT) and several charge-density based approaches employed, we aimed at addressing several fundamental questions. Is the distance criterion (H···I < 3 Å) a necessary and sufficient condition for the assignment of hydrogen bonding between H and I atoms in o-CH_3_NH_3_PbI_3_? If the answer is yes, are the intermolecular bond distance cut-offs generally used for conventional C···H, O···H, N···H, S···H, F···H, and Cl···H hydrogen bonds^62–64^ pertinent for I···H hydrogen bonds, given that the van der Waals radii^65^ of C (1.77 Å), O (1.50 Å), N (1.66 Å), S (1.89 Å), F (1.46 Å) and Cl (1.82 Å) are substantially different from that of I (2.04 Å)? Are significant noncovalent interactions limited to only polar bonds, such as the N‒H bond in the NH_3_^+^ moiety of MA (methylammonium)? If this is so, does this lead to the exclusion of putative interactions involving the C‒H low polarity bonds of the ‒CH_3_ moiety? If interactions involving the latter are not insignificant, do they contribute to controlling the tilting of PbI_6_^4−^ octahedra in o-CH_3_NH_3_PbI_3_ and can they be quantified energetically?

In other terms, we shall show that I···H–C hydrogen bonds should not be overlooked as they partly contribute in determining the overall structure of o-CH_3_NH_3_PbI_3_. We shall also address the interesting question of whether hydrogen bonding controls octahedral tilting, as demonstrated previously, or whether it is the tilting that dictates the pattern of noncovalent interactions. We shall present evidence that intermolecular distance cut-off (a tentative approximation!) alone cannot be used to determine whether or not there is an attractive intermolecular interaction between two atoms in a complex system. While it may well be that on occasion intermolecular interactions at distances >3 Å fall into the weak bonding regime, they are certainly not necessarily inconsequential for the bulk structure of materials^62–64,66–71^, as well as that for supermolecular molecular systems in the gas phase.

This paper is organized as follows. In the following section, we provide the details of computational methodologies adopted, including the reasoning why they were chosen. Following this is the Results and Discussion section, where we explore chemical bonding scenarios addressing the questions raised above using various state of the art computational approaches, including comparison between the accuracy of lattice constants evaluated theoretically using standard DFT methods with those known experimentally. In a subsection of the latter, we provide a description of strength of the importance of the organic cation and its possible role in the tilting of the PbI_6_^4−^ octahedra of the inorganic framework. Finally, we summarize our results in the Conclusion section.

## Computational Methods

Most of the periodic DFT calculations reported to date on CH_3_NH_3_PbI_3_ have presented the patterns of chemical bonding and its physical chemistry based on a static geometry, even though the organic cation is subjected to significant dynamics around room temperature and even at 25 K^72^. The reliability of the calculated structure has been inferred from a comparison with available X-ray or neutron diffraction structures^38,73^. As noted previously^31,36^, it is not straightforward to provide the nature of bonding in halide perovskite crystals using X-ray diffraction structures since it is difficult to correctly assign the position of N, C and H atoms within the framework of heavy atoms such as I and Pb; nor it is easy to provide a definitive conclusion on the cation dynamics since there is a lack of appropriate agreement between theoretical and experimental data, which is due to the large spread in the outcomes of the different experimental reports that make it difficult to judge the accuracy of any particular level of theory^74^. What is known about the orientation of the ammonium and methyl groups of the organic cation inside the perovskite cage at and above room temperature is nothing other than an approximation since the cation experiences rotational dynamic motion, and was inferred from spectral signatures on the average (static) geometries at different snapshots and from chemical intuition. Taking this into account, we have used the more reliable neutron diffraction structures of o-CH_3_NH_3_PbI_3_ and o-CD_3_ND_3_PbI_3_ for benchmarking DFT geometries even though these were determined at temperatures > 0 K, and are representatives of metastable ground states of the system.

The PBE (Perdew-Burke-Ernzerhof) exchange-correlation functional^75,76^, together with a *Γ*-point centered 10 × 8 ×10 *k*-point mesh for Brillouin zone sampling, as in previous studies^77,78^, and a plane-wave energy cut-off of 520 eV, was used to obtain the relaxed bulk geometry of o-CH_3_NH_3_PbI_3_. The Projector Augmented Wave (PAW) method^79,80^ was utilized for the relaxation of ionic positions and unit-cell parameters. The tolerance for total energy convergence was set to 10^−5^ eV atom^−1^ rather than the default setting. The average and maximum intermolecular forces on ions after relaxation were below 0.005 and 0.009 eV Å^−1^, respectively (all below 0.01 eV Å^−1^), and the stresses were below 0.05 Gpa; similarly as in other studies^17,24,25,32^. VASP^81–84^, the Vienna Ab initio Simulation Package, was used.

Regardless of whether one uses Density Overlap Regions Indicator (DORI)^85^, Reduced Density Gradient (RDG) noncovalent index^86^, or quantum theory of atoms in molecules (QTAIM), all provide insight into chemical bonding provided the geometry of the chemical system under investigation is well defined; this is because these approaches are all geometry dependent, as are any other properties of the system (such as polarity, for example). The Gaussian 09 package^87^ was therefore used for the generation of wavefunctions, with the PBE, or PBE + vdW, or neutron diffraction geometry supplied. PBC single points were performed and an all-electron correlated double-ζ DZP basis set obtained from the EMSL basis set library^88^ was invoked. These were used for the evaluation of the RDG, DORI, and QTAIM based electron density properties associated with the coordinate and noncovalent bonding interactions. Depending on the evaluation of specific properties and visualization, software packages such as Critic 2^89^, AIM-UC^90^, Multiwfn^91^, AIMAll^92^, and VMD^93^ were used.

Before proceeding to the Results and Discussion section below, we answer the following questions raised by some anonymous reviewers: Will the absence of (i) Spin-Orbit Coupling (SOC) and ii) vdW corrections affect the DORI analysis^85^ and other results of the o-CH_3_NH_3_PbI_3_ system? We answer question i) as follows. SOC does not determine the geometry of the halide perovskite system since it not a determinant of the geometry of the system; rather it affects the electronic properties. This view is in agreement with Whalley and coworkers^94^. Moreover, it must be kept in mind that relaxation of the geometry of the system with SOC significantly increases the computational timescale, providing no remarkable changes to the local geometry^74,95^ that could affect intermolecular interactions^23,96–99^. If the importance and nature of band dispersion and non-equilibrium quantum transport properties are the main concern of a study, a SOC calculation is necessary^100^. It assists in an understanding of the nature of the splitting of the valence and conduction band extrema at the band edge, called valence band maximum (VBM) and conduction band minimum (CBM), respectively, by which the CBM is generally and appreciably affected, causing the character of the bandgap (*E*_*g*_) to change from direct to indict or *vice-versa* for organic-inorganic hybrid halide perovskites. However, this is not the case for o-MAPbI_3_ since the bandgap is always direct at the *Γ*-point. This is because SOC does not cause any splitting of either of the band extrema, although the magnitude *E*_g_ is affected. The decrease in the magnitude of the *E*_g_ due to SOC can be as large as 1 eV^94,101^, which is due to the degenerate (empty) 6p orbitals that are split and shifted apart in energy. This attribute has been referred to as a structure-dependent SOC response, which is enhanced for less tilted structures leading to an effective bandgap reduction^102^. We note that this (direct-to-indirect bandgap transition) attribute has been reflected in the structure of the same MAPbI_3_ system in the cubic phase. In this phase, the orientation of the organic cation inside the cage is believed to lie along three possible directions, (100), (111) and (110), as well as along their other equivalent directions (*viz*. (0 ± 11), (01 ± 1), (±101), (±110), (10 ± 1), (01 ± 1)). Because of this, the structure of the system is locally affected. When the effect of SOC is taken into account in the analysis of band dispersion, the direct to the indirect character of the bandgap is generally encountered with respect to the orientation of the organic cation, which is due to the band splitting and the shift of the CBM from *R* → *M* in *k*-space. This has been explained as a result of the intermolecular hydrogen bonding interactions between the I atoms and MA that are not symmetric, which affects symmetry of the overall system, causing the space group of the system to transit from *Pm3m* to *P1* in the relaxed geometry^94^, and is indeed not the subject of this study. Large band dispersion generally leads to high electron- and hole-mobility, and consequently, to large diffusion length for the charge carriers, which is good for efficient charge carrier extraction^103^. These discussions widely scattered in the perovskite literature might explain why the majority of theoretical studies performed SOC single points on the energy-minimized geometry of the MAPbI_3_ system.

To answer question (ii), we refer to the study of Li and Rinke^37^. These authors have suggested that hydrogen bonding, which is well described by the PBE functional, plays a decisive role in the structural parameters of these systems, including the position and orientation of the organic cation as well as the deformation of the inorganic framework. The vdW-induced lattice-constant corrections are system-dependent and PBE + vdW lattice constants are expected to be in good agreement with experiment. As we show in the following section below, the inclusion of the effect of vdW does not reproduce exactly the experimental structures, i.e., the intermolecular interactions could not be significantly magnified by incorporating vdW corrections, yet it significantly influences the unit-cell volume compared to that calculated using PBE without vdW. We have indeed observed marginal changes on the lattice constants, and other geometrical properties (bond distances and bond angles, *etc*.), but this did not lead to any change in the overall conclusions on the nature and type of intermolecular interactions arrived at by incorporating vdW corrections. We have previously observed an analogous result for the CH_3_NH_3_PbBr_3_ system by performing vdW corrections at different levels of theory^3^. It is, however, worth noting that vdW plays a major role especially when one is solely interested in the determination of the stabilization energy of an intermolecular interaction; this is not the case here since it is not possible to determine exactly such an energy of the individual intermolecular interactions involved between the inorganic host and the organic guest using periodic DFT calculations, because there are many of them that are immersed in the interaction of the amine and methyl H atoms with the iodide inorganic host. Nevertheless, the effect of van der Waals (vdW) correction was considered at the DFT-D3 level (vdW-D3) with Becke-Jonson damping^104,105^ as incorporated in VASP^81–84^.

Since SOC has no direct influence on the structure, its exclusion should not affect the results of DORI, QTAIM and RDI based NCI approaches. Since the vdW correction has some effect on the overall geometry, lattice parameters and intermolecular interactions, it would have some impact on the strength of the interactions; what effect this does have on these interactions in o-CH_3_NH_3_PbI_3_ is discussed below.

## Results and Discussion

**Figure 1:**
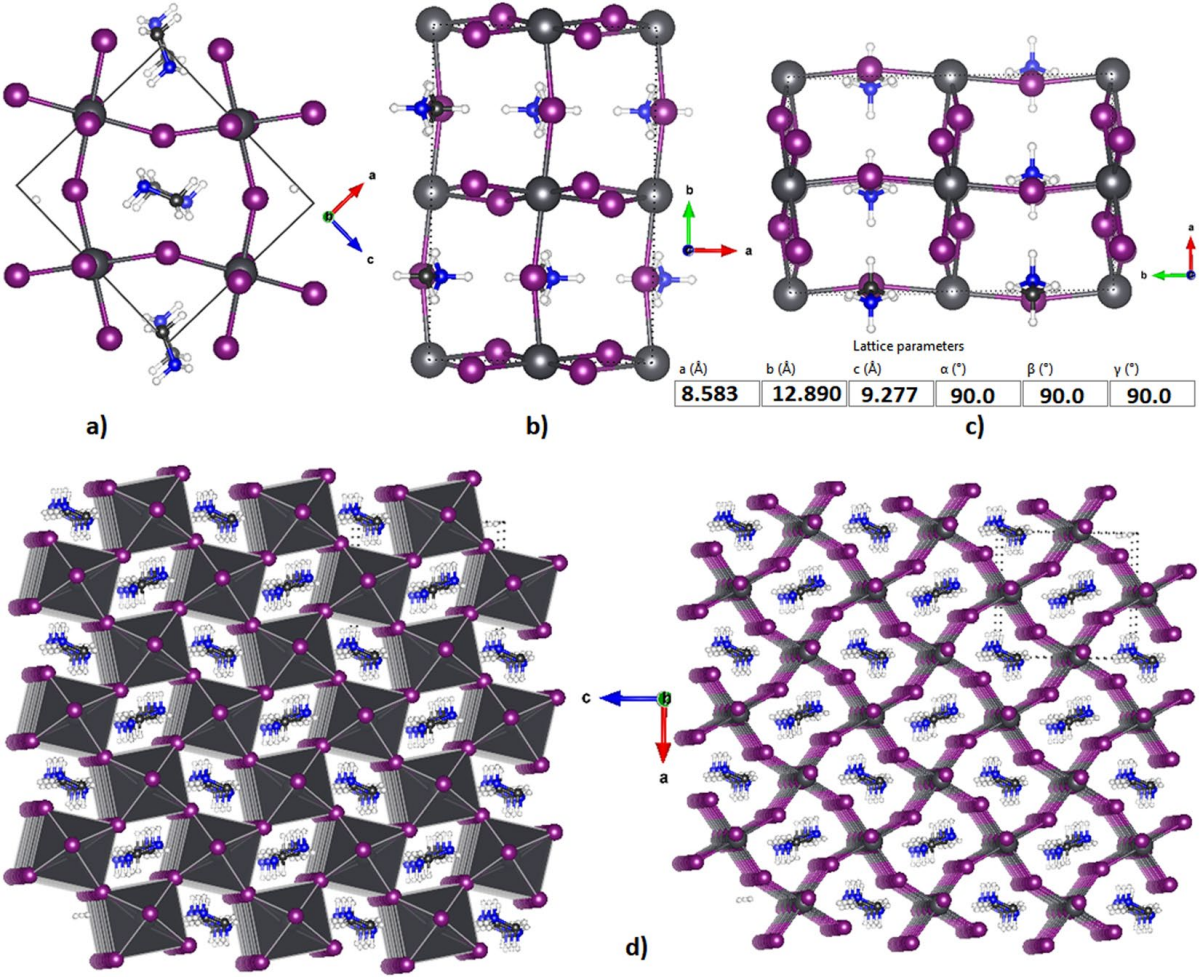
(**a**–**c**) Ball-and-stick views of the unit-cell of o-MAPbI_3_ (48 atoms). In (**b**,**c**), the I atoms outside the cell boundary are not shown. (**d**) The polyhedral and ball-and-stick display of the 3 × 3 model of o-MAPbI_3_, showing the the supramolecular (thin film type) geometry of the infinite crystal emerges from the repetition of the unit-cell only. PBE optimized lattice constants for the unit-cell are shown in c).

**Figure 2:**
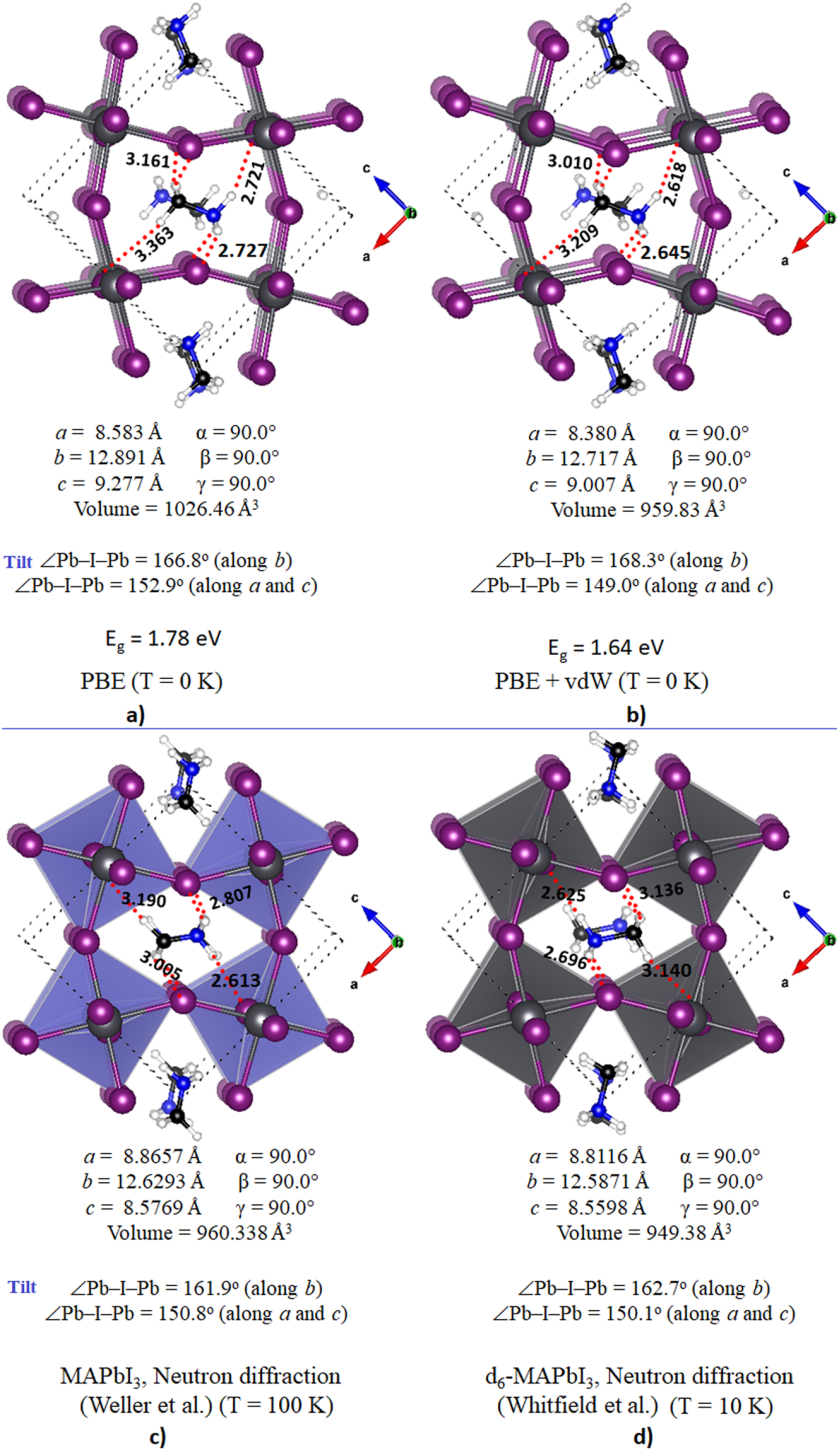
Comparison of (**a**) PBE and (**b**) PBE + vdW calculated unit-cell properties of o-CH_3_NH_3_PbI_3_ with those of (**c**) o-CH_3_NH_3_PbI_3_ (T ≈ 100 K)^31^ and (**d**) d_6_-CD_3_ND_3_PbI_3_ (T ≈ 10 K)^32^ reported using neutron diffraction measurements. Included are the lattice constants, tilt angles and unit-cell volumes. Calculated bandgaps with and without vdW corrections are shown for the calculated geometries. Temperatures T are indicated.

The various representations of the PBE relaxed unit-cell geometry of o-CH_3_NH_3_PbI_3_ are shown in Fig. 1a–c), and the periodic extension of the unit-cell in all three directions is illustrated in Fig. 1d). The optimized lattice constants are shown in Fig. 1c. Although the PBE computed lattice constant *c* is about 0.7 Å larger than that reported for o-CH_3_NH_3_PbI_3_ (*T* ≈ 100 K)^31^ and b) CD_3_ND_3_PbI_3_ (*T *≈ 10 K)^32^ using neutron diffraction measurements (Fig. 2), all other lattice constants were well reproduced. There is a marked difference in the unit-cell volumes when the calculated and experimental values are compared (Fig. 2). Moreover, the calculated bandgap, which is the energy difference between the top of the valence band maximum and the bottom of the conduction band minimum, is 1.78 eV for o-CH_3_NH_3_PbI_3_.

The inclusion of a vdW correction has some impact both on the bond distances and bond angles, and caused a reduction in all three lattice constants. The volume of the unit-cell was also affected significantly, reducing from the PBE value of 1026.46 Å^3^ to the PBE + vdW value of 959.83 Å^3^, in which the latter is closer to the experimentally reported value of 560.338 Å^3^ (Fig. 2). A consequence of this is that the bandgap, which was calculated to 1.78 eV for the uncorrected structure, was reduced to a value of 1.64 eV after vdW correction.

The bandgap values noted above are in reasonable agreement with the experimental value of 1.65 eV (4.2 K)^106^. We note furthermore that the PBE bandgap matches that of 1.81 eV reported previously with the SOC-GW approach^106^. It also matches with that of 1.63 eV reported with HSE06^77,78^, but is smaller than values of 2.07 and 1.96 eV reported with GGA and PBE + D_2_, respectively^77^. The 3×3 model of o-CH_3_NH_3_PbI_3_ has a very marginal effect on both the geometries (e.g., bond distances and angles) and bandgap; hence the unit-cell geometry and its properties are a good representation of these properties of the supramolecular composite structures. Thus DFT modeling of the properties of the bulk geometry can be used as an approximation for the understanding of any large-scale system, as discussed in other studies^107,108^.

Figure 2c,d show the unit-cell geometries and lattice constants for o-CH_3_NH_3_PbI_3_^31^ and o-CD_3_ND_3_PbI_3_^32^ from neutron diffraction measurements. We have used the o-CD_3_ND_3_PbI_3_ geometric data for this study as the C–H bond lengths of the –CH_3_ group in MA of o-CH_3_NH_3_PbI_3_ are reported to be longer than the corresponding C–H and C–D distances of several deuterated analogues of the same system reported in the orthorhombic phase^32^. For instance, the C–H bond lengths in o-CH_3_NH_3_PbI_3_ (*T* = 100 K) range between 1.215 and 1.114 Å^31^, which are significantly longer than the C–H/C–D bond distances in o-CD_3_NH_3_PbI_3_ (1.078–1.088 Å at 10 K), o-CH_3_ND_3_PbI_3_ (1.083–1.091 Å at 10 K), o-CD_3_ND_3_PbI_3_ (1.072–1.083 Å at 10 K) and o-CD_3_ND_3_PbI_3_ (1.030–1.027 Å at 130 K)^32^. This shows that the I···H–C/ I···D–C intermolecular distances between the perovskite host lattice and the organic guest in the orthorhombic polymorphs reported in the two studies are different.

There are two possibilties that explain the somewhat longer C–H bond lengths in o-CH_3_NH_3_PbI_3_. First, this could be due to the H atom positions that were determined at a higher temperature, i.e., an effect of temperature. Second, and because of this, there may be partial proton transfer between the I and H(C) atoms; the proton transfer phenomena are, in general, associated with bond elongation and concomitant red-shifting in the vibrational frequency of the covalently bonded proton (H) linked with a Lewis base in an intermolecular interaction. Moreover, even though o-CD_3_ND_3_PbI_3_ is fully deuterated, the presence of D species should hardly cause any marked elongation to the C–D compared to the C–H bond in a given compound. Because of the larger atomic mass of D, its involvement in a noncovalent interaction, in principle, makes the interaction stronger since the zero-point vibrational motion of the C–D bond plays an important role. This is not unreasonable given that the quantum mechanical description of the C–D system compared to the C–H system shows the former has a relatively lower zero-point energy, which presumably corresponds to the minimum energy configuration that the quantum system can attain. The zero-point motion causes the former to vibrate more slowly than the latter due to the difference in their masses that affect the stabilization energy of the intermolecular interaction that they individually form with a given negative site; the former needs more energy for bond dissociation than the latter. This feature emerged from many experimental studies; it is well established, and thus requires no further explanation^109–111^.

Nevertheless, and in contrast with what has been suggested in^25^, simply because the N···I distances are shorter than the C···I distances and the C–H···I angle is smaller than the N–H···I angle is not grounds for ignoring possible C–H···I hydrogen bonds. N···I distances must necessarily be shorter than C···I distances given that N has a smaller van der Waals radius than C (1.66 *vs*. 1.77 Å^65^). There is enough precedence^62–64,66,67^ that in the case of C···H hydrogen bonds the angle of approach of the electrophile ranges between 90 and 180° and the intermolecular distance is usually smaller than 3.30 Å^112^; the latter is significantly larger than the sum (2.97 Å) of the van der Waals radii of C and H. This is in accordance with Torshin *et al*.^113^ who have proposed a number of empirical rules to define a minimal geometrically-consistent set of the criteria for the identification of a P···H−A hydrogen bond (P is the proton acceptor species, and A is the proton donor fragment covalently bonded to atom H).

The neglecting of possible I···H–C interactions, as was done in some reports^24,25^ and many other experimental studies, is at odds with a more recent study^17^. In this, it was shown that both the Br···H–C and Br···H–N hydrogen bonds have significant strength and together explain the tilting of the PbBr_6_^4−^ octahedra in the related perovskite, o-CH_3_NH_3_PbBr_3_. In particular, it was found that the Br···H–C interactions become significant in the low temperature orthorhombic polymorph. This observation was used to rationalize the state of the MA cations and their effect on the concomitant tilting of PbBr_4_^‒^ octahedra, with a consequent dynamic change in the band structure. A similar finding was recently reported by us for the same system^3^, providing further evidence of the reliability of the I···H–C interactions in the o-CH_3_NH_3_PbBr_3_ perovskite system. The possibility of such close contacts in CH_3_NH_3_PbI_3_ has also been pictorially indicated in many other other studies such as in the report of Ong *et al*.^114^, but not discussed.

There are many experimental studies that suggest that if two atoms of opposite charge from two molecular species are in closer proximity than the sum of their vdW radii, then there is the possibility of the formation of a significant noncovalent intermolecular interaction between them^62–64,66–70,115^. As the van der Waals radius of I is 2.04 Å and that of H/D is 1.20 Å^65^, any I···H intermolecular distance <3.24 Å in o-CH_3_NH_3_PbI_3_ signifies a potentially significant intermolecular interaction. This same argument also applies to I···D–N and I···D–C distances in o-CD_3_ND_3_PbI_3_ that have the d_6_-MA species inside the inorganic cage (Fig. 2d).

**Figure 3:**
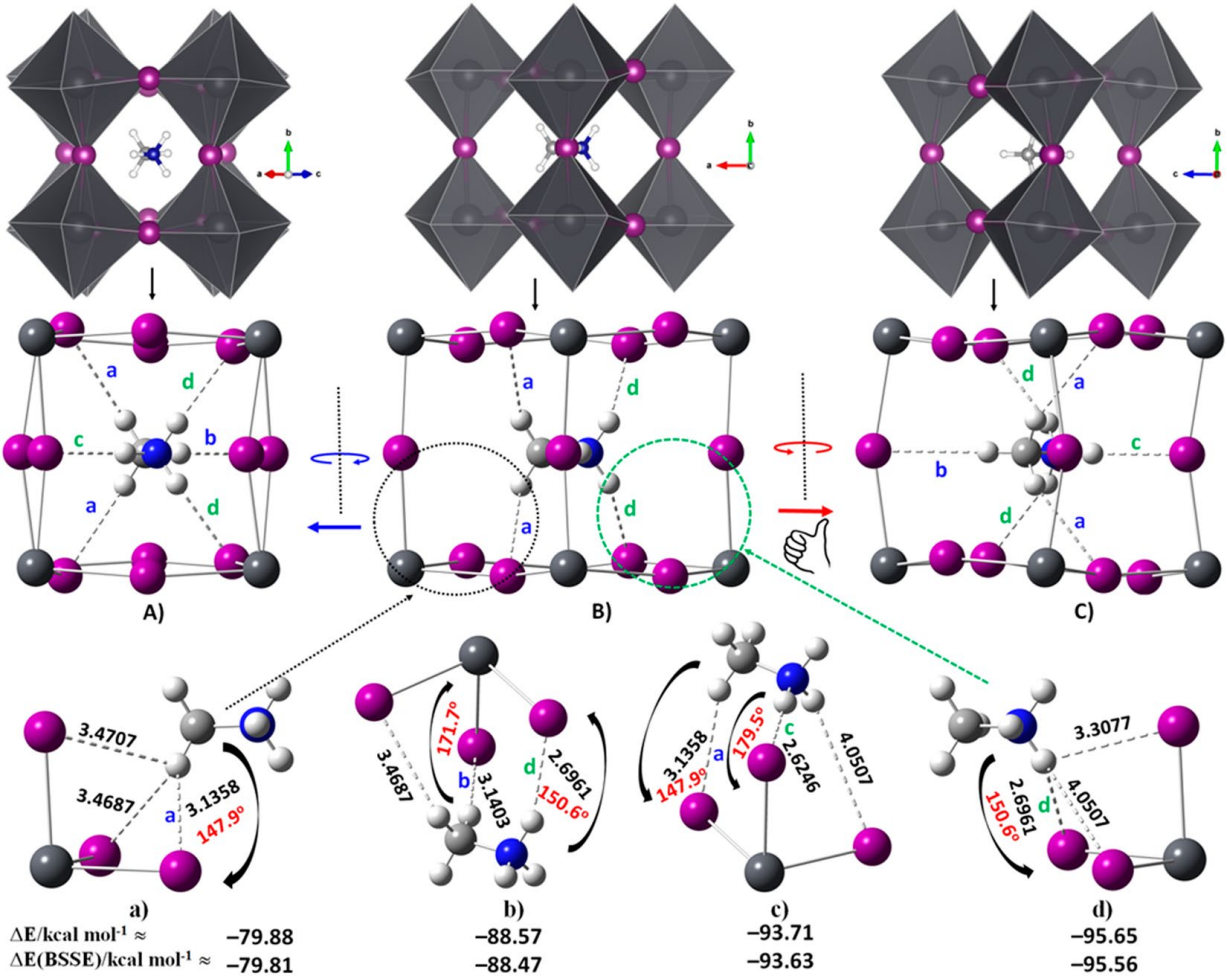
Polyhedral (upper) and ball-and-stick (middle) model views of o-CD_3_ND_3_PbI_3_ (10 K) from powder neutron diffraction data^32^. The distances marked a, b in blue and c, d in green in (**a**) through (**c**) distinguish between the potential I···D–C and I···D–N deuterium bonds. The a and d labels are repeated for equivalent bonds. Similar hydrogen bonded contacts are identifiable in the structure reported for crystal data of o-CH_3_NH_3_PbI_3_^31^, although with somewhat different intermolecular distances (Fig. 2). Shown in (**a**–**d**) are the CD_3_ND_3_^+^···^-^I_3_Pb molecular blocks extracted from the polymorph B) (see text for detail).

The ball-and-stick and polyhedral views presented in Fig. 3 show a variety of snapshots of the organic species inside the cage of o-CD_3_ND_3_PbI_3_. For instance, the geometry B) of the system leads to the snapshots shown in A) and C) through approximately clockwise and anticlockwise rotations of the overall geometry of the system, respectively. Based on intermolecular bond distances between the host and guest species (Fig. 2), one might identify three I···D–C and three I···D–N (possible) interactions in the experimental geometry of o-CD_3_ND_3_PbI_3_^32^, marked a-b and c-d, respectively; there is a similar possibility in the experimental geometry of o-CH_3_NH_3_PbI_3_^31^. For instance, the I···H–C intermolecular distances available in the supplementary information of o-CH_3_NH_3_PbI_3_ (Fig. 2c) show two interactions at 3.005 Å and one at 3.190 Å; the corresponding I···H–N distances are 2.807 Å(×2) and 2.613 Å, respectively. These distances are all <3.24 Å, consistent with the range for hydrogen bonds formed by the C–H donor^116^. There is therefore mutual penetration between the I and H atomic basins, indicative of the presence of a hydrogen bond^117–119^. However, an experimental increase in the temperature of the system increased the I···H(–N) bond lengths such that these were in the ranges 3.15–3.18 Å and 3.12–3.52 Å at 180 K and 352 K, respectively; this is associated with the change of phase of the system. Because spread between the experimental data for bond lengths is so large, it is difficult to make the best choice between the computational methods and their accuracies, in concordance with other views^74^.

The PBE calculated bond distance values are 2.727 vs 2.721 Å for I···H(–N), and 3.161 vs. 3.363 Å for I···H(–C) contacts, showing some discrepancy especially in the latter values compared to the neutron diffraction data. Incorporating a vdW correction resulted in a slight decrease in these bond distance, *viz*., 2.645 and 2.618 Å for the fomer and 3.010 and 3.209 Å for the latter (Fig. 2). Even so, there is no significant difference between either of these calculated geometries compared with that observed between the two types of I···H(–N) bonds in either of the crystal geometries. While the simulation cell volume was found to change significantly with the vdW correction, which also occurs on changing the nature of the correlated level^74^, this did not markedly affect the intermolecular interaction distances.

Lahnsteiner *et al*.^74^ have carried out molecular dynamics simulation in the temperature range 250 K to 400 K with several DFT functionals. Their results show Gaussian probability distribution for the I···H–N and I···H–C hydrogen bonds. For example, the former bonds were found with values in the range 2.75–2.80 Å with PBE, and 2.75–2.79 Å with PBEsol, and have observed an increase of these distances when passing from T = 250 K through 300 K to 350 K to 400 K. An opposite trend was found for the I···H–C hydrogen bonds over the same temperature range. The underlying reason for this behavior of the bond distances was unclear from the study, but this could be due to the mobile nature of the H atoms of the amine group undergoing proton transfer as the temperature of the system increases.

In 1998, Steiner summarized some experimentally reported I^−^···H and I^−^···C/N distances in I^−^···H–C/N hydrogen bonds that have ∠I^−^···H–C/N < 140°^120^. The distances for I^−^···H–C(Cl_3_), I^−^···H–C(H_2_Cl_2_), I^−^···H–C(NN), and I^−^···H–C(CN) were 2.84 and 3.86 Å, 2.85 and 3.88 Å, 2.90 and 3.85 Å, and 2.99 and 4.00 Å, respectively. Similarly, the I^−^···H(N) and I^−^···N distances in crystals involving –NH_3_^+^, N^+^H_2_, (CC)N^+^H and I^−^ were 2.72 and 3.68 Å, 2.76 and 3.61 Å, 2.63 and 3.58 Å, respectively, thus showing the N–H donors to form relatively shorter intermolecular contacts than C–H donors as expected based on our arguments using vdW radii. Moreover, while the existence of X···H–C hydrogen bonding was a matter of controversy between 1937 and 1995^62,121–123^, Aakeröy and coworkers^124^ in 1999 pointed out that the chemical and biochemical community tend to ignore, or are unaware of, such interactions, or, of more concern, dismiss them as insignificant. As they pointed out, this is unfortunate since such hydrogen bonds are of great importance in molecular recognition processes, the reactivity and structure of biomolecular species, the stability of complexes, crystal engineering, molecular conformation and ionic liquids. While the sum of the vdW radii, as a meaningful selection criterion for hydrogen bonding, is mainly electrostatic in origin, an attractive force which does not decrease greatly with increasing distance is thus still expected to be significant well beyond the vdW separation. The sum of the van der Waals radii to validate intermolecular hydrogen-bonding interactions is a very poor criterion. These authors especially noted the importance of the aforesaid interactions as a prototype and argued that Cl···H–C hydrogen bonds might be present in a wide range of compounds containing heterocyclic aromatic systems. Although Desiraju believed that the Cl···H–C interaction was still questionable at that time, others recognised its existence; this eventually led to a demonstration that the phenomenon is universal, and not restricted to any specific category of compounds. It was further shown that the Cl^−^···H–C interaction is an attractive hydrogen bonding interaction, commonly displayed at distances greater than the conventional vdW limit, often occurring between 2.9 and 3.1 Å. C–H hydrogen bonds to fluorine, bromine and iodine were demonstrated to show similar patterns of bonding. This is in line with the concluding remarks of Taylor^125^: “It took me 30 years to be persuaded that C−H···F−C and C−H···Cl−C contacts matter, but I remain of this view. Whether this belief is accepted by others or not, one thing is clear: any explanation of the crystal packing of the structures discussed herein must account for the fact that they contain many more X···H interactions than would be expected by chance”.

A statistical potential developed by Jiang and Lai^126^ has quantitatively described the O···H(C) hydrogen bonding interaction at the protein-protein interface. The calculated energies of the O···H(C) interaction pairs showed a favorable valley at about 3.3 Å^126^, exhibiting the feature typical of a hydrogen bond; this is similar to the *ab initio* result reported by others elsewhere^127^. These authors have demonstrated that the low polarity C–H bond has the potential to engage in an attractive interaction to form a hydrogen bond. Similarly, as Jeffery noted a few decades ago^128^, the proximity between a polyhydroxy molecule and a protein they examined does involve hydrogen bonding, contrary to prevailing views. It is, in the context of current thought, also likely that the I···H(C) hydrogen bonds in o-CH_3_NH_3_PbI_3_ have not been recognized as possible hydrogen bonded synthons that contribute to its structural development in the low and room temperature phases. Studies of F···H–C, Cl···H–C, Br···H–C, I···H–C and C–H···π furnish further evidence concerning the significance of intermolecular interactions that can be formed by the low polar C–H donor^125,129–134^.

The immediate question that arises now is how to quantify the strength of these hydrogen and deuterated bonded interactions in o-CH_3_NH_3_PbI_3_ and o-CD_3_ND_3_PbI_3_, respectively. The answer is not straightforward because there is no simple way available to calculate the stabilization (binding) energy of either of these hydrogen bonds in o-CH_3_NH_3_PbI_3_, or of the deuterium bonds in o-CD_3_ND_3_PbI_3_. This is because the bulk structures are modeled theoretically using periodic boundary conditions, containing 48 atoms with different numbers of PbI_3_^−^ and CH_3_NH_3_^+^ fragments. Another reason is that intermolecular interactions involved between the organic and inorganic cores are all collective, and are responsible for the overall stability of the geometry of the system. Estimation of the stabilization energy might be easier for the cubic geometry of the system since the CH_3_NH_3_PbI_3_ bulk in the cubic phase is composed of a single PbI_3_^−^ subunit and a CH_3_NH_3_^+^ subunit. Again, in doing so, one would use a periodic calculation for the PbI_3_^−^ lattice to estimate total electronic energy, and a similar calculation for the cation in a large box centered at *Γ-*point to evaluate the total electronic energy of CH_3_NH_3_^+^. The difference between the sum of these energies and the total electronic energy of the CH_3_NH_3_^+^I_3_Pb^−^ system would provide the stabilization energy of the system comprising several I···H–N hydrogen bonds^19^. However, due to symmetry reasons, this would not give a realistic estimation of the magnitude of the binding energy of an individual I···H–N interaction. Thus, in order to quantify approximately the binding energy of the intermolecular interaction formed by the H atom(s) of the –NH_3_^+^ and –CH_3_ fragments of MA with the perovskite cage in o-CH_3_NH_3_PbI_3_, or of the –ND_3_^+^ and –CD_3_ fragments of d_6_-MA with the same cage in o-CD_3_ND_3_PbI_3_, we have listed in the bottom panel a)–d) of Fig. 3 four CD_3_ND_3_^+^···^−^I_3_Pb binary configurations as examples. Each of these represents a local geometry in o-CD_3_ND_3_PbI_3_ (Fig. 3b). For instance, the blocks a) and d) represent the two local intermolecular geometries of the periodic geometry B), which are indicated by black and green dotted circles, respectively. The construction of each of these blocks was made possible by keeping the local CD_3_ND_3_^+^···^−^I_3_Pb molecular block stoichiometry and deleting the remaining Pb and I atoms of the o-CD_3_ND_3_PbI_3_ cuboctahedron illustrated in Fig. 3b; the rationale for this is as given above, that is, CH_3_NH_3_PbI_3_ in the room temperature cubic phase can be regarded as being made up of CH_3_NH_3_^+^···^−^I_3_Pb ion-pairs. Nevertheless, all the intermolecular distances between the inorganic and organic fragments that are marked by the dotted lines in each molecular block in Fig. 3b do not necessarily represent an intermolecular hydrogen bonded interaction, but are shown to clarify whether such a possibility exists within the framework of the bulk configuration. The intermolecular bond angles are shown only for the unambiguous contacts (see below for further discussion).

We used the Gaussian 09^87^ package and the PBE/Def2-TZVPPD method to explore the nature of the binding energy. The triple-ς quality Def2-TZVPPD pseudopotential basis set accessed from the EMSL basis set exchange library^88^ was used. The actual crystal environment may have some influence on the binding energy, but, as indicated above, this is a compromise since there are no available theoretical methods to provide insight into this. While calculating the binding energy of the cluster models, we did not ignore the importance of the monopole-monopole term; one should not ignore its influence since any intermolecular interaction is driven by Coulombic forces; in addition, the extent of the exchange-repulsion, dispersion and polarization effects determine the overall magnitude of the intermolecular interaction at equilibrium. This is the subject of several discussions^135,136^.

Single points at the PBE/Def2-TZVPPD level of theory were carried out on each of the four block geometries illustrated in (a–d) of Fig. 3. This has enabled the determination of the uncorrected and basis set superposition error (BSSE)-corrected binding energies (Δ*E* and Δ*E*(BSSE), respectively). As such, the Δ*E* was calculated by subtracting the sum of the total electronic energies of the two monomers interacting with each other (PbI_3_^−^ and CD_3_ND_3_^+^) from the total electronic energy of the CD_3_ND_3_^+^···^−^I_3_Pb ion-pair complex. Addition of Δ*E* to the energy due to the BSSE results in Δ*E*(BSSE).

We found Δ*E*(BSSE) = −79.81 kcal mol^−1^ for block conformation a), and −95.56 kcal mol^−1^ for block conformation d). The former cluster involves I···D–C intermolecular contacts, whereas the latter involves the I···D–N intermolecular contacts. The large binding energies are not surprising given these complex systems involve attractive engagements between oppositely charged species leading to charge neutrality of the building blocks, driven predominately by electrostatics with appreciable contributions from dispersion and polarization interactions (see below)^18^. Thus block conformation a), which involves purely I···D–C intermolecular contacts, has a binding energy that is significantly more negative than −40 kcal mol^−1^, the so-called covalent limit for hydrogen bonding^63,64^, and is a consequence of more than a single interaction between the interacting fragments.

The binding energy analysis signifies that the I···D–C interactions are competitive with the I···D–N deuterium bonds in o-CD_3_ND_3_PbI_3_, even though these are weaker than the latter. This feature is in qualitative agreement with NMR findings (see below), as well as with many discussions that have advanced the same view. The same argument applies to the I···H–C and I···H–N hydrogen bonds in o-CH_3_NH_3_PbI_3_ since the block models of CH_3_NH_3_^+^···^−^I_3_Pb corresponding to the clustered configurations a), b), c) and d) of Fig. 3 extracted from the crystal geometry^31^ of o-CH_3_NH_3_PbI_3_. These four geometries gave comparative values of −79.49 [−79.42], −87.74 [−87.66], −95.79 [−95.71] and −97.04 [−96.96] kcal mol^−1^ for Δ*E*[Δ*E*(BSSE)], respectively.

Correction for dispersion is nowadays regarded as an essential component for computing geometries, and PBE itself does not do that. Similar calculations as above were therefore performed for the four blocks of CD_3_ND_3_^+^···^−^I_3_Pb with Coupled Cluster Theory at CCSD(T)/Def2-TZVPPD. The Δ*E*[Δ*E*(BSSE)] calculated at this level of theory were −84.66 [−81.25], −92.82 [−89.13], −97.07 [−93.79] and −99.75 [−96.26] kcal mol^−1^ for configurations a), b), c) and d), respectively (Fig. 3). While PBE somewhat underestimates the binding energies, it correctly predicts the physical trend expected for the electrostatically dominant noncovalent interactions between the binary cluster models examined. That the hydrogen bonds in organic-inorganic perovskites are strong is not unusual since these bonds can well be typified as (double) charge-assisted hydrogen bonds.

It is worth mentioning that we have recently discussed whether gas phase calculations are useful in getting at least some information on the nature of the stability of iodine-centered intermolecular interactions^4,19^. We found that the nature of the intermolecular interactions and the overall geometries obtained with a gas phase calculation can be used to infer what happens in the crystalline phase. In any case, the binding energies calculated using the solid state local geometries shown in Fig. 3 and those determined previously on the gas phase optimized structures^4^ did not differ markedly from one another; the observed difference can be attributed not only to the intra- and inter-molecular geometries obtained using periodic and non-periodic DFT calculations, but also to the orientational degrees of freedom of the monomers in the clusters and the basis sets of different qualities that were used in these two studies.

For comparison, binding energies in the range between −77.94 and −106.99 kcal mol^−1^ for the [C(NH_2_)_3_]^+^[Mn(HCOO)_3_]^−^ and [(CH_2_)_3_NH_2_]^+^[Mn(HCOO)_3_]^−^ metal-organic perovskites have been reported^137^, highlighting that “small bonds [are] a big deal in perovskite solar cells”^138^ and in this case, the impact of the monopole-monopole term was seemingly included. Binding energies of comparable magnitude have been reported by for single hydrogen bonds and or single halogen bonds in Y^−^···^+^YX (Y, X = F, Cl, Br, I) and Y^−^···^+^HD systems^139^. Cation-anion hydrogen bonds, with binding energies ranging from −80 to −210 kcal mol^−1^, have been reported as a new class of hydrogen bonds that extends their strength beyond the covalent limit^140^. These hydrogen bonds have a strength comparable to charge assisted halogen bonds as reported only recently^141^. Energies of similar magnitude were reported by Vradwaj *et al.*^4–6^ and Alkorta *et al*. in a recent study of perovskite clusters^142^.

The cluster model (Fig. 3) in many cases mimics the real nature of the intermolecular interactions in crystals^19,107^, but further information about the reliability of these interactions can be obtained from a quantum theory of atoms in molecules (QTAIM) analysis^143^. This was carried out both on the calculated and experimental unit-cell geometries of the o-CH_3_NH_3_PbI_3_ and o-CD_3_ND_3_PbI_3_ systems. The mathematical and physical aspects of the theory are now well-established^144^, albeit that discussions concern the interpretation of some quantities derived from QTAIM are on-going. It has been applied to help delineate the nature of the chemical bonding in a wide range of chemical systems^54,145–154^ and is especially useful in systems which lack a simple reference bonding model.

### QTAIM description of bonding

**Figure 4:**
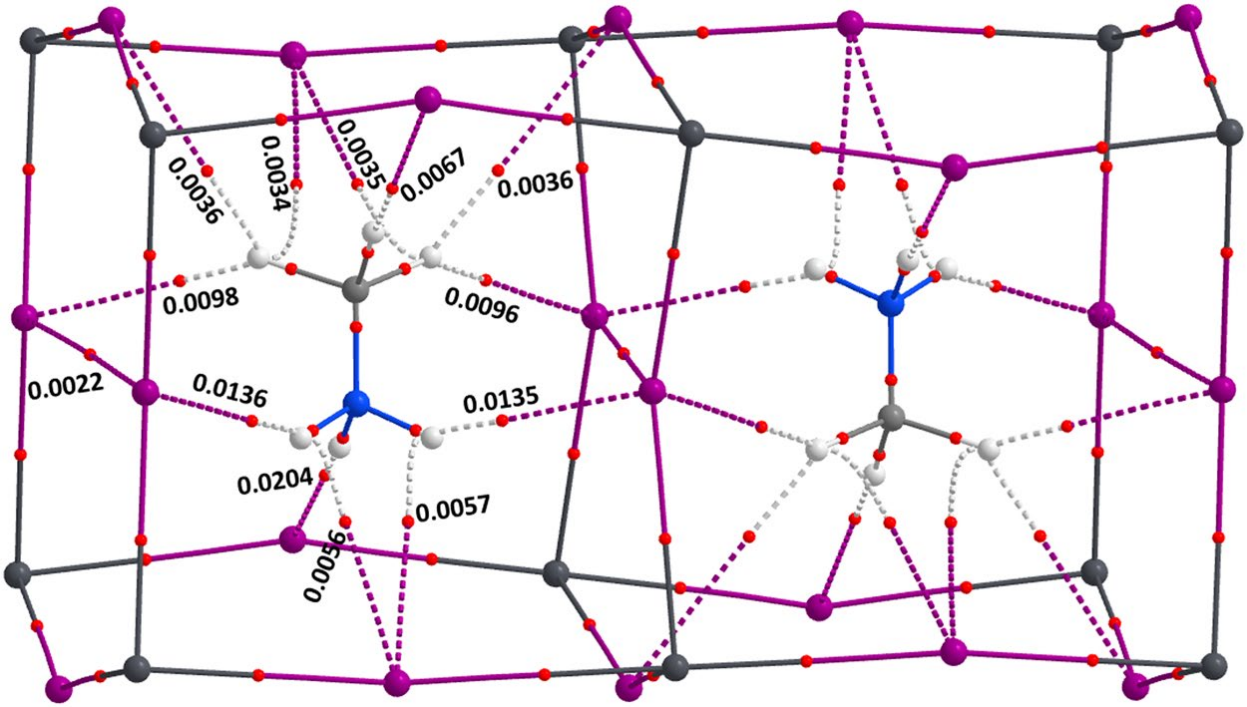
PBE QTAIM molecular graph and (3, −1) bond critical point charge densities (au) for o-CH_3_NH_3_PbI_3_, obtained on its neutron diffraction geometry (T ≈ 100 K)^31^. Bond paths are shown as solid and dotted lines in atom colour and (3, −1) bond critical points as tiny red spheres. Atoms are illustrated as large spheres, with iodine: purple; carbon: dark-grey; nitrogen: deep-blue; and hydrogen: white-grey.

**Figure 5:**
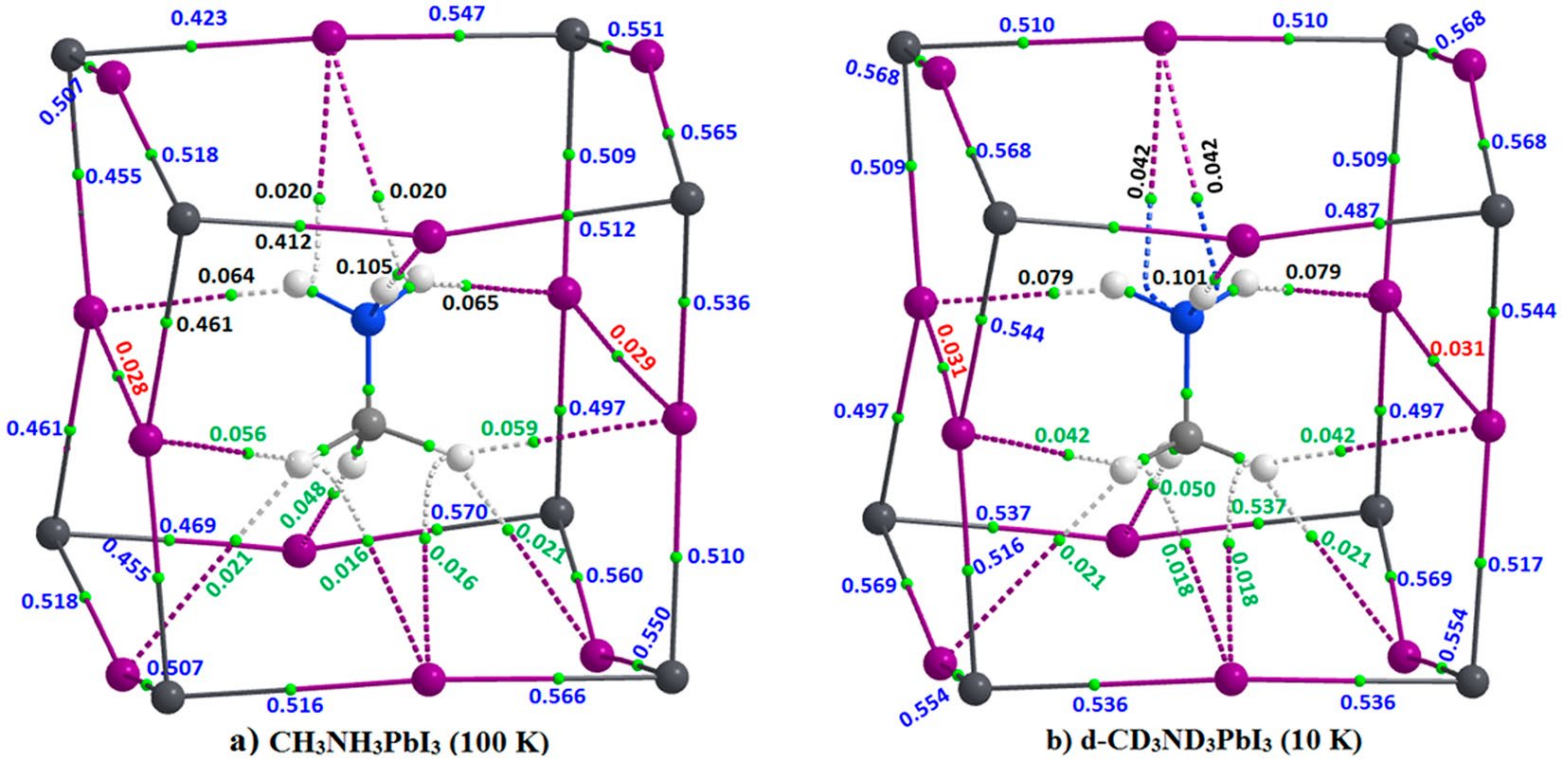
PBE QTAIM molecular graphs for (**a**) o-CH_3_NH_3_PbI_3_ (T ≈ 100 K)^31^ and (**b**) o-CD_3_ND_3_PbI_3_ (T ≈ 10 K)^32^. The values shown are the delocalization indices δ for various atomic pairs. Bond paths are shown as solid and dotted lines in atom colour and (3, −1) bond critical points as tiny green spheres. Atoms are illustrated as large spheres, with iodine: purple; carbon: dark-grey; nitrogen: deep-blue; and hydrogen: white-grey. Numbers in various colors refer the type of bonding interactions that discriminate from one another.

The PBE level QTAIM molecular graph evaluated on the experimental geometry of o-CH_3_NH_3_PbI_3_ is shown in Fig. 4. A similar result was obtained for o-CD_3_ND_3_PbI_3_ as well (Fig. 5b). The topological presence of bond paths and bond critical points (bcps) of charge density observed between bonded atomic basins is in agreement with IUPAC recommendations^54,154^. In other words, each C–H bond of the cation MA is attractively engaged with one or more iodides of the inorganic framework, forming I···H(‒C) contacts. As shown in Fig. 4, each H atom of −NH_3_^+^ is involved in the formation of either a single or a bifurcated hydrogen bonding interaction. By contrast, each H atom of –CH_3_ in MA is involved in the formation of either a single or a trifurcated hydrogen bonding interaction. A similar bonding pattern also emerged for o-CD_3_ND_3_PbI_3_ (Fig. 5). Details of the charge density at various bond critical points in o-CH_3_NH_3_PbI_3_ are displayed in Fig. 4.

From the discussion above, one can conclude that these I···H(/D)‒N and I···H(/D)‒C interactions collectively and simultaneously contribute to the development of the highly symmetric *Pnma* architecture of o-CH_3_NH_3_PbI_3_/o-CD_3_ND_3_PbI_3_ perovskites in the low-temperature phase, and that the tilting of the PbI_6_^4−^ octahedra are a delicate balance between the competition of both types of hydrogen bonds and the distortion caused by lattice forces. However, from this result, as well from the energies of these interactions given above, it is rather difficult to determine which specific interaction is predominantly responsible for tilting of the octahedra in a specific direction, although the charge density bond critical point properties signify I···H(/D)‒N to be relatively stronger than I···H(/D)‒C.

In addition to the two types of hydrogen bonding interactions discussed above, the QTAIM analysis suggests that there are two near-equivalent I···I interactions in o-CD_3_ND_3_PbI_3_/o-CH_3_NH_3_PbI_3_. These could be regarded as lump-hole interactions^155^, but are largely dispersive. Longer interaction distances of this type were reported in [C_6_H_5_NH(CH_3_)_2_]_2_Te_2_I_10_^156^ with I···I distances of 3.66 and 3.80 Å, shorter than twice the van der Waals radius of I (4.04 Å). These contacts have been interpreted as promoting charge carrier migration throughout the Te—I network. Whether these noncovalent interactions are real is addressed below.

Furthermore, from a molecular electrostatic surface potential analysis^157^, we found that the outer surfaces of the C and N atom of MA are positive. Hence, the inwardly curved bond paths between the outer electrostatic surface of the carbon atom of the –CH_3_ fragment of MA and the I atom of the inorganic perovskite cage in Fig. 4 could be regarded as a consequence of π-type carbon (tetrel) bonding interaction since a p-type anti-bonding orbital of the N–C bond is accepting electron density from the coordinated iodide atom with which it is interacting^158–161^. A similar conclusion can be arrived at for the bent bond paths developed between the outer electrostatic surface of the N atom of –NH_3_^+^ in MA and the I atoms of the perovskite cage illustrated in Fig. 4; these signify the presence of π-type pnictogen bonding^161,162^. An identical conclusion can be arrived at from the molecular graph of o-CD_3_ND_3_PbI_3_ (Fig. 5b).

An important result from QTAIM is the delocalization index, δ^143,149–153,163–165^, which is a measure of covalent bond order^163–165^. It is this property that can be evaluated for any pair of atomic basins in molecular systems or in crystals even when there is no evidence for a bond path and a (3, −1) bcp between them. Because it is as a measure of the electron-pair sharing between two atoms A and B, it should be related to the character (single, double, triple) of the bond. It has been used to categorize hydrogen and dihydrogen bonds regardless of whether these are weak or strong^166^, and to classify coordination bonds in transition metal complex systems^167^. The electron-pair density δ for a given pair of atoms A and B for closed-shell systems is represented by one-electron density matrix given by Eqn (1)^168–170^, where *S*_*ij*_(*A*) and *S*_*ij*_ (*B*) are orbital overlaps integrated within the basins of atoms A and B, respectively, and the summation run over all occupied spin-orbitals.

1 $$\delta (A,B)=4\sum _{i}^{occ}\sum _{j}^{occ}{S}_{ij}(A){S}_{ij}(B)$$

The δ values for some important atomic pairs in o-CH_3_NH_3_PbI_3_ and o-CD_3_ND_3_PbI_3_ are given in Fig. 5. These are in the range 0.45−0.60 for Pb—I, 0.064−0.105 for I···H(N), 0.021−0.059 for I···H(–C), and 0.028−0.029 for the I···I bonding interactions in o-CH_3_NH_3_PbI_3_ (Fig. 5a). Similar values of δ were determined for the corresponding atomic pairs involving the D atom in o-CD_3_ND_3_PbI_3_ (Fig. 5b). Comparable δ values have been reported previously for analogous bonding interactions. For example, δ = 0.0236 for H_2_BH···HBr^166^; 0.50 < δ < 1.23 for M—C; and 0.04 < δ < 0.22 for M—O bonds for some transition metal carbonyl complexes^171^. The results summarised in Fig. 5 suggest that the various coordination and intermolecular bonding interactions identified and characterized for o-CH_3_NH_3_PbI_3_ and o-CD_3_ND_3_PbI_3_ are not ambiguous, and therefore should contribute, each to some extent, to the overall structures of these systems.

### A DORI description

There are two other electron density approaches often ulilized to provide insight into the chemistry of noncovalent interactions. One of these is the DORI approach^85^; the other is the Reduced-Density-Gradient Noncovalent Interaction (Index) (RDG-NCI) approach^86^. Another theoretical approach, the Electron Localizability Indicator (ELI-D)^172^, has also been employed for this purpose, but we did not use it in this study. While DORI has been suggested to produce results that are complementary to those obtained from QTAIM^143^, ELI-D^172^, and RDG-NCI^86^ approaches, it has an advanage that, in addition to providing information on covalent interactions, it can also provide insight into the chemistry of noncovalent interactions. We have also employed both DORI and RDG-NCI approaches to explore the nature of the chemical bonding topologies involved between various atomic basins in o-CH_3_NH_3_PbI_3_^31^.

**Figure 6:**
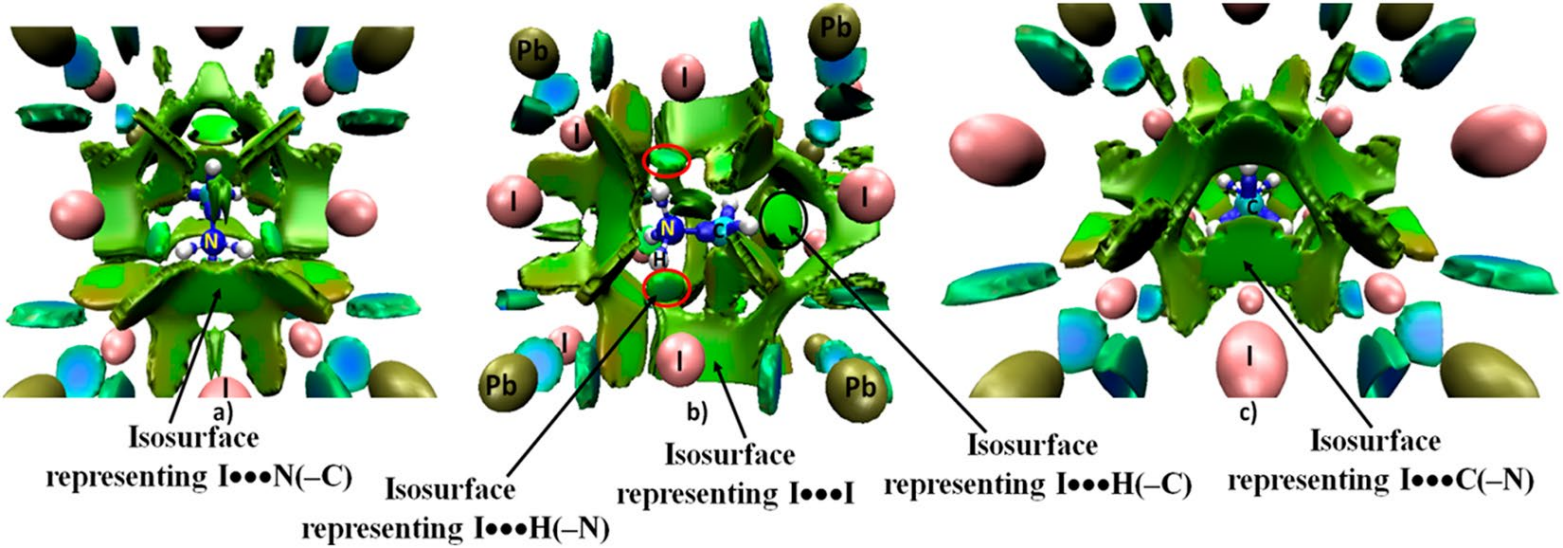
Various views (**a**–**c**) of the PBE level density overlap regions indicator (DORI = 0.95 au) isosurfaces of o-CH_3_NH_3_PbI_3_^31^. Labeling of atom type is shown in (**b**). The broad deep-green isosurface between N of –NH_3_^+^ and I of PbI_6_^4−^ in (**a**) indicates the presence of I···N–C pnictogen bonding, whereas that between C of –CH_3_ and I of PbI_6_^4−^ in (**c**) indicates the presence of I···C–N carbon bonding. The disc-like circular isosurfaces in (**b**) represent the I···H–N and I···H–C interactions; a few of these are marked by red and black ellipses, respectively. The isosurfaces in blue appearing between the C and N atomic basins, between the N and H atomic basins, and that between the C and H atomic basins in CH_3_NH_3_^+^ represent regions dominated by covalent bonding. The disc-like isosurfaces coloured blue-green appearing between the Pb and I atoms represent dative coordinate bonding interactions. The remaining broad and irregular isosurfaces around the organic cation are probably the consequence of the presence of some secondary van der Waals type interactions between the organic cation and the I atoms of the perovskite cage interior.

As can be seen from Fig. 6, the volume of each of the DORI isosurfaces associated with a I···H(–C) interaction is somewhat larger than, or comparable to, that of a I···H(–N) interaction. It shows this former interaction is somewhat more dispersed than the latter, giving further evidence that the I···H(–C) interaction is not insignificant. Clearly, the three I···H(–N) interactions alone cannot be used to describe the tilting of the PbI_6_^4−^ octahedra in o-CH_3_NH_3_PbI_3_ as has been done by others^25^. This is because the two equivalent and one longer I···H(–C) bonds in o-CH_3_NH_3_PbI_3_ that are formed between the –CH_3_ moiety of MA and the cage iodides (*vide supra*) are also well linked with the *a*^*−*^*b*^+^*a*^*−*^ octahedral tilting of the PbI_6_^4−^ octahedra.

As with QTAIM, DORI has identified all the I···I contacts in o-CH_3_NH_3_PbI_3_; they are manifest as broad, dispersed and somewhat irregular semi-circular isosurfaces occurring between the interacting I atoms (Fig. 6b). The covalent bonds of the CH_3_NH_3_^+^ cation are shown through the appearance of blue isosurfaces between the interacting atomic basins within its molecular framework, which are due to significant delocalization of electron density in the bonding regions.

DORI reveals the presence of I···N(–C) and I···C(–N) pnictogen- and carbon-bonding interactions (see Fig. 6a,c, respectively), as well as several other van der Waals-type interactions between the organic and inorganic frameworks. The latter are not unexpected given that the cationic organic guest species is hosted in an anionic cage.

These results suggest that the tilting of the PbI_6_^4−^ octahedra in o-CH_3_NH_3_PbI_3_ is driven by the joint involvement of various noncovalent interactions, including I···H(–C), I···H(–N), I···N(–C), I···C(–N), and I···I, among others. These results give an indication that all these interactions collectively responsible for *the extent of tilting* of the PbI_6_ octahedra in o-CH_3_NH_3_PbI_3_.

### Reduced density gradient analysis

**Figure 7:**
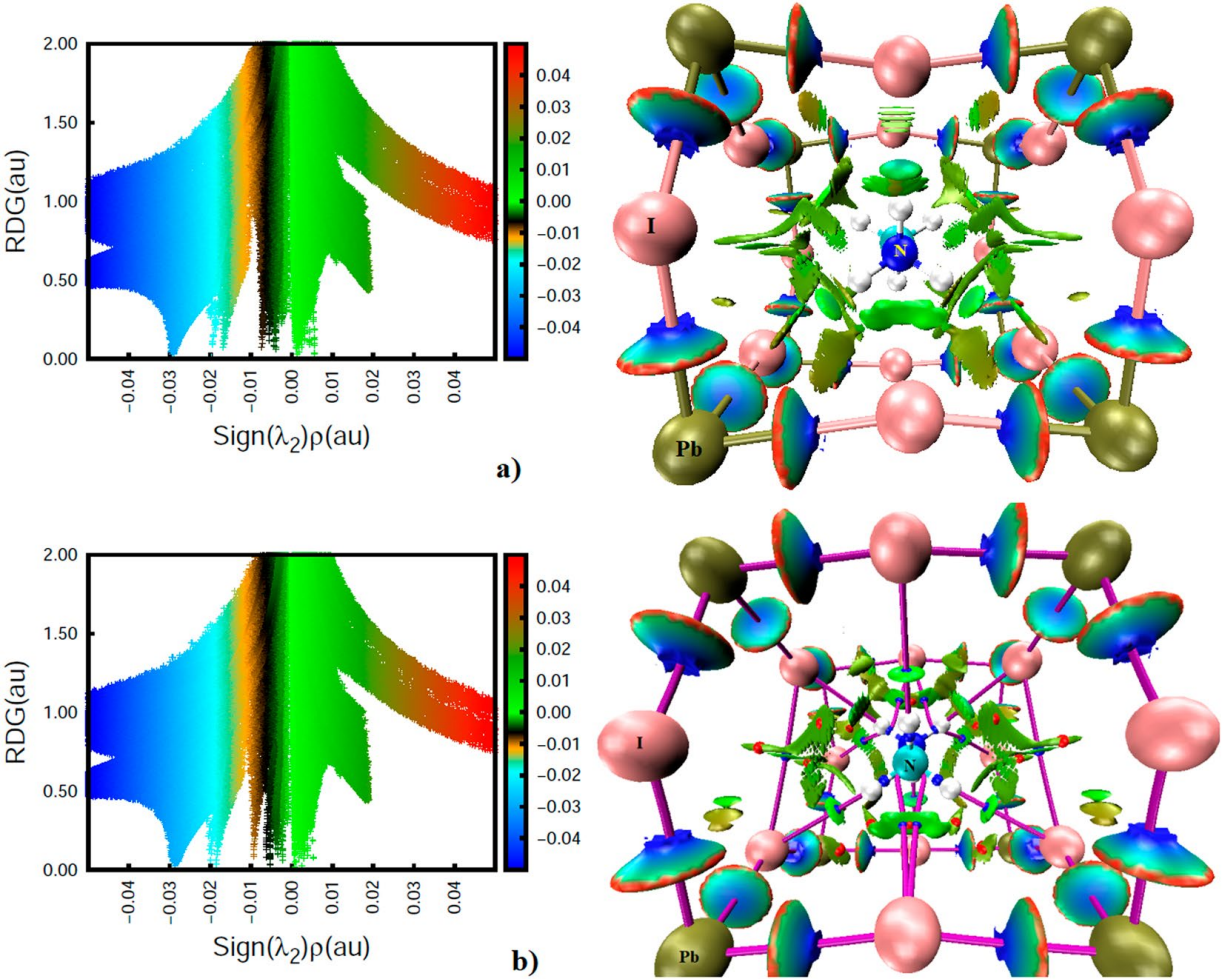
PBE level sign(λ_2_) × *ρ* vs. RDG (left) and isosurface (right) NCI plots, showing the presence of various noncovalent bonding interactions between the perovskite host and guest (MA/d_6_-MA) species in (**a**) o-CD_3_ND_3_PbI_3_ (neutron diffration geometry, 10 K) and (**b**) o-CH_3_NH_3_PbI_3_ (DFT-D3 geometry). Atom type is shown. The coloring scheme in sign(λ_2_) × *ρ* vs. RDG was chosen to assist in distinguishing the amplitude of the electron density corresponding to different types of interactions. The isosurfaces on the right are colored such that blue, cyan, and green isosurfaces represent very strong, strong, and medium-to-weakly bound interactions, respectively, whereas those colored in red represent repulsive interactions. Bond- and ring-critical points in (**b**) are shown as tiny spheres in blue and red, respectively, whereas bond paths are shown as solid lines in purple.

**Figure 8:**
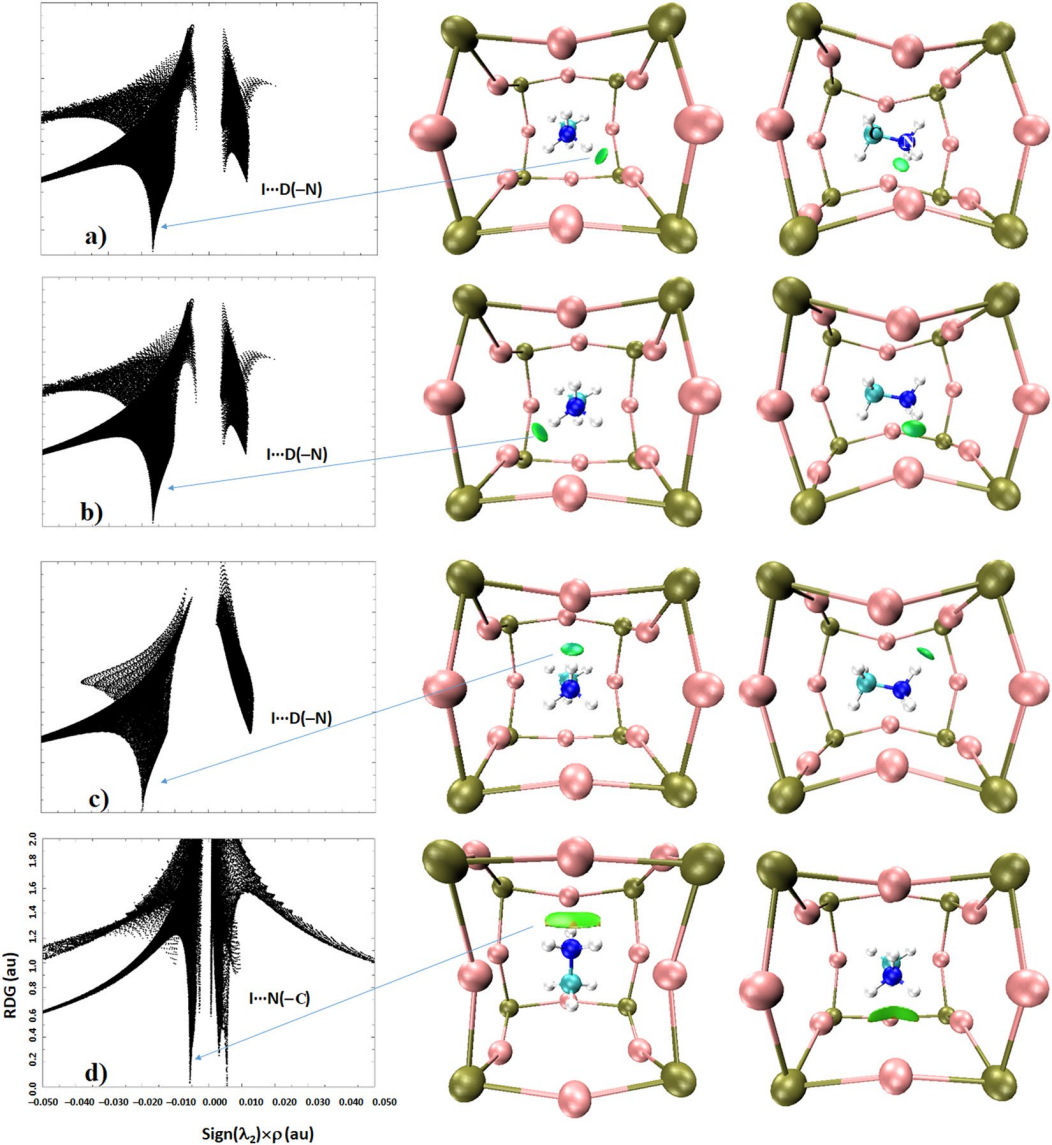
(**a**–**d**) Views of PBE level RDG NCI plots showing some dominant I···D(–N) and I···N(–C) noncovalent bonding interactions, obtained on the reported neutron diffraction crystal geometry of o-CD_3_ND_3_PbI_3_ (10 K). Left: sign(λ_2_) × *ρ* vs. RDG (au); right: 3D RDG isosurface domains (isovalues 0.56 au). Atom labeling is given in Fig. 7a.

**Figure 9:**
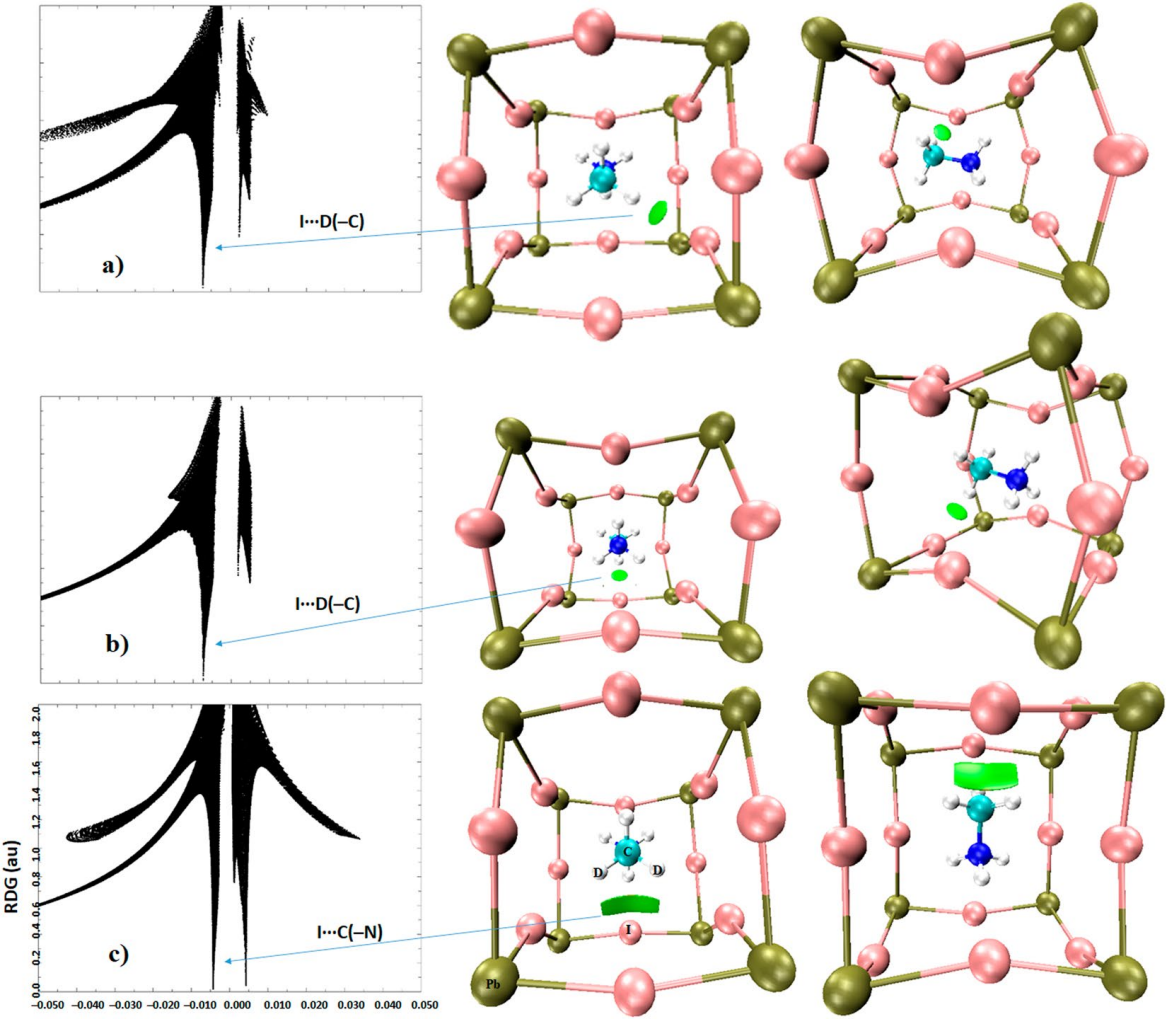
(**a**–**c**) Views of PBE level RDG NCI plots showing some potential I···D(–C) and I···C(–N) noncovalent bonding interactions, obtained on the reported neutron diffraction crystal geometry of o-CD_3_ND_3_PbI_3_ (10 K). Left: sign(λ_2_) × *ρ vs*. RDG (au); right: 3D RDG isosurface domains (isovalues 0.56 au). Atom labeling is given in Fig. 7a.

The RDG based NCI results are illustrated in Figs 7–9. These were obtained on the experimental geometry of o-CD_3_ND_3_PbI_3_ (10 K); similar results were obtained for o-CH_3_NH_3_PbI_3_ and are shown in part in Fig. 7b. As is evident in Fig. 6, the 3D isosurface plot of DORI analysis is complex since the presence of several intermolecular interactions increases the extent of overlapping of various DORI domains in the plot. One of the possible reasons for this is that DORI uses a relatively large isovalue of 0.95 au, which when decreased below 0.5 au, for instance, generally leads to the disappearance of most of the DORI domains. That the DORI plot is complicated was also the opinion of many anonymous reviewers, who have come up with the same view that an RDG analysis could be a better choice for the characterization of noncovalent interactions as it may make the isosurface plot clearer. We note, however, that the complexity of the RDG domains emanating from this analysis is comparable to that of the DORI domains (Fig. 7) if both analyses are carried out with the same isovalue of 0.95 au. Even so, and as indicated already above, the only advantage of RDG analysis is that this does not show up the RDG domains between the covalently bonded atoms expected within the covalent framework of MA.

To avoid the difficulties just mentioned, we carried out an RGD analysis for individual atom-atom interactions to confirm that the QTAIM- and DORI-predicted interaction topologies correctly represent the attractive interactions identified. The RDG results between the various atomic domains that potentially dominate the interaction between the ammonium fragment of the organic cation and the inorganic anionic cage in o-CD_3_ND_3_PbI_3_ are given in Fig. 8. The first three graphs (Fig. 8) show that there are disc-shaped RDG volumes between the I and D atoms (colored blue-green). The coloring scheme indicates that the electron density in the bonding region is not significantly depleted. This is a general coloring feature of any RDG analysis, and is a characteristic of the region of space described by λ_2_ < 0, where λ_2_ is the second (diagonal) eigenvalue of the Hessian second derivative charge density matrix. Since this provides an indication of the presence of an attractive interaction, we conclude that the I···D(–N) deuterium bonds in o-CD_3_ND_3_PbI_3_ as characterized by QTAIM and DORI do indeed exist.

The bottom graph of Fig. 8 shows a relatively flat and broad RDG volume between the –ND_3_ fragment and the I atom facing it. The appearance of such a broad volume suggests that the electron density distribution in the bonding region is a consequence of the involvement of several atoms. These include simulteneous interactions of D and N atoms of the –ND_3_ with the closest iodide atom of the inorganic cage. The isosurface, colored in green, indicates that the electron density in the bonding region is somewhat depleted relative to that found between the I and D atoms reponsible for the I···D(–N) deuterium bonds (Fig. 8a–c). However, the dominant attractive contribution arises from the N atom of the –ND_3_ fragement since its electrostatic surface is deficient of electron density and interacts with iodide to form an I···N(–C) pnictogen bond. This result concurs with the QTAIM bond path topology that shows up between N and I atoms (Fig. 4). The presence and strength of this, as well as the I···D(–N) deuterium bonds in Fig. 8a–c, accord with the RDG spikes of the sign(λ_2_) × *ρ vs*. RDG graphs plotted in 2D. For instance, the two equivalent and longer I···D(–N) bonds correspond to the region −0.020 au < sign(λ_2_) × *ρ* < −0.018 au and the remaining shorter I···D(–N) bond corresponds to the region −0.018 au < sign(λ_2_) × *ρ* < −0.016 au in the sign(λ_2_) × *ρ vs*. RDG graphs; both regions signify the presence of potential hydrogen bonds.

Figure 9 shows the results of the RDG analysis perfomed between individual atoms of the –CD_3_ fragment of d_6_-MA and the iodides of the inorganic perovskite cage. Not all intermolecular interactions are shown, and for clarity, only the dominant ones are presented. In essence, the first two of these (from top) represent one longer and two equivalent I···D(–C) deuterium bonds, respectively; the corresponding intermolecular distances are shown in Fig. 2. The isosurfaces representing these are colored in green because the distribution of the electron density around the I and D(–C) bond critical point regions is weaker than those of the I···D(–N) deuterium bonds. This can be inferred by comparing the spikes characteristic of the λ_2_ < 0 region in the sign(λ_2_) × *ρ vs*. RDG plots in 2D, that is, in the region −0.008 au < sign(λ_2_) × *ρ* < −0.004 au. These results further confirm that it is not just the iodides that move to the D(–N) moieties to form I···D(–N) bonds. The same conclusion can be arrived at for the I atoms involved in the I···D(–C) bonds. However, such a movement for I···D(–N) is relatively larger than that for I···D(–C). This effect is noticeable if one carefully scrutinizes the corner shared I atoms that are responsible for the I···D(–N) and I···D(–C) interactions (see the I atoms linked with the C–D fragments in Fig. 9a,b, for example).

Analogous to the RDG isosurface found between I and N(–C), characterized above as pnictogen bonding, there is a broad and flat isosurface between C and I (Fig. 9c (bottom)). The RDG spike, with a negative sign(λ_2_), that appears in the region −0.010 au < sign(λ_2_) × *ρ* < −0.007 au provides the strength of this interaction. Another spike appears in the region +0.003 au < sign(λ_2_) × *ρ* <  +0.005 au, which provides evidence that there are other van der Waals interactions between the −CD_3_ fragment and iodide, which probably explains the reason for the broadness of the RDG volume between them. This result is consistent with the QTAIM bond path topology found between the −CD_3_ fragment and the interacting iodide, forming the local C–H–I–H ring structure. Curved bond paths responsible for this are normally present in systems where classical arguments predict the presence of strain, as for example in small ring hydrocarbons^173^. The bent nature of the bond paths towards the interior of the ring is not surprising since it appears to present a maximized binding interaction from a minimum amount of electron density^173^.

**Figure 10:**
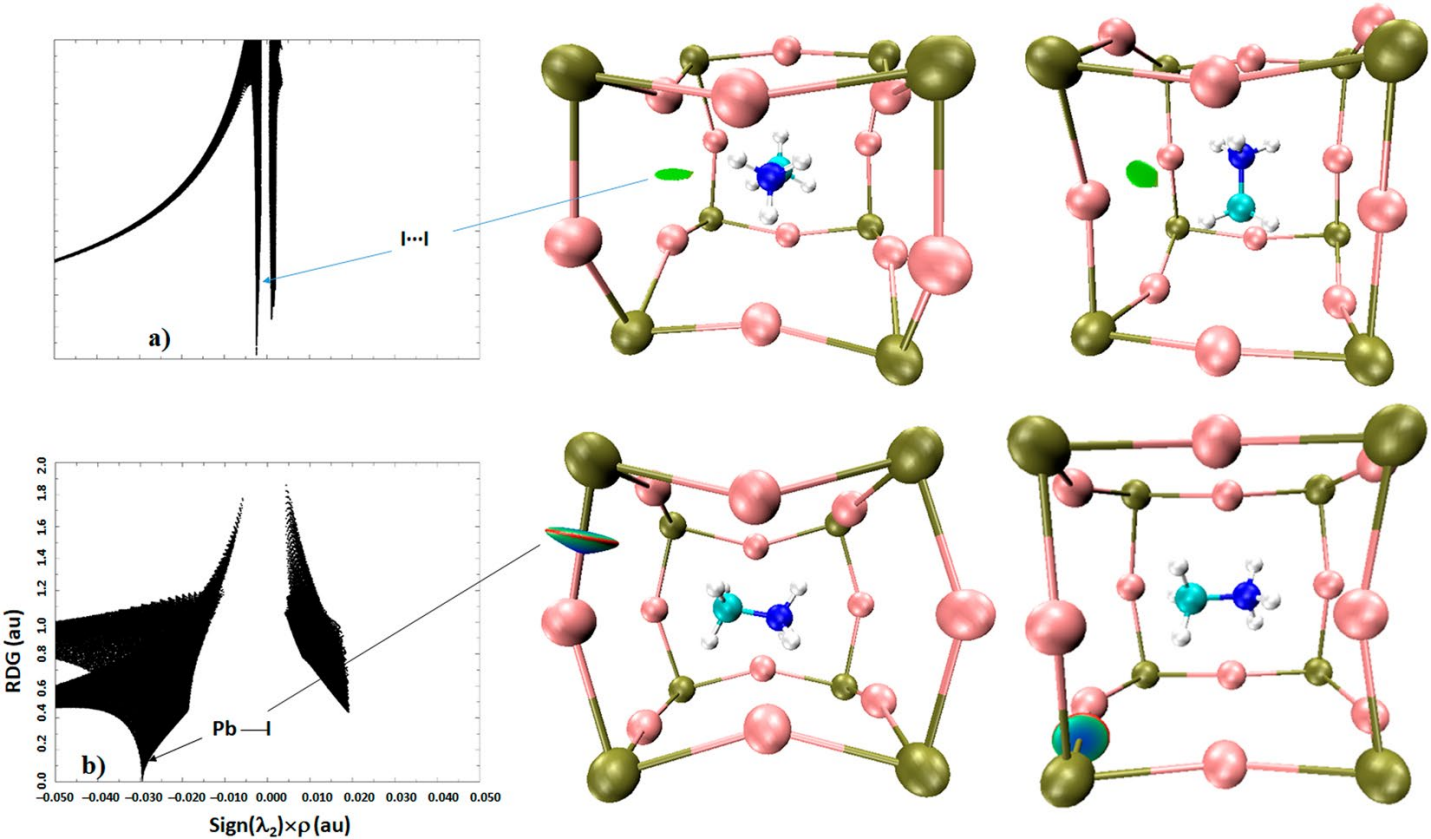
(**a**–**b**) Views of PBE level RDG NCI plots showing some I···I (top) and Pb—I (bottom) van der Waals and dative coordination bonding interactions, respectively, obtained on the reported neutron diffraction crystal geometry of o-CD_3_ND_3_PbI_3_ (10 K). Left: sign(λ_2_) × *ρ* vs. RDG (au); right: 3D RDG isosurface domains (isovalues 0.56 au). Atom labeling is given in Fig. 7a.

Figure 10 confirms that I···I noncovalent interactions revealed using QTAIM and DORI analyses are not a computational artefact. They are the inherent geometrical features of the o-CD_3_ND_3_PbI_3_ system and are dispersive. This nature is evidence of both the RGD spikes appearing in the regions −0.007 au < sign(λ_2_) × *ρ* < −0.005 au and +0.001 au < sign(λ_2_) × *ρ* <  +0.003 au, which correspond to the RDG isosurface domains colored green.

It is worth mentioning that Pb—I dative coordinate bonding interactions in o-CD_3_ND_3_PbI_3_ and o-CH_3_NH_3_PbI_3_ are not purely ionic interactions. Coordinate bonds comprise both covalent and ionic components (*vide infra*)^74^. In agreement with this, the RDG plot illustrated in Fig. 10b (bottom-left) shows significantly more delocalization of electron density at the Pb and I critical bonding region, which is reflected in the RDG isosurface that is significantly more bluish-cyan than green. In the sign(λ_2_) × *ρ vs*. RDG plot, this coordinate bonding is described by the region −0.028 au < sign(λ_2_) × *ρ* < −0.032 au. We note that Lee and coworkers attributed the −0.015 au < sign(λ_2_) × *ρ* < −0.030 au region in their study to I···H(–N) hydrogen bonding for o-CH_3_NH_3_PbI_3_^24^, although this region comprises both I···H(–N) hydrogen bonding and Pb—I coordinate bonding interactions. A similar analysis was performed by El-Mellouhi and coworkers^50^; their sign(λ_2_) × *ρ vs*. RDG plot for o-CH_3_NH_3_PbI_3_ matches with ours for I···H(–N) hydrogen bonding. Surpringly, the presence of other spikes corresponding to the weak-to-medium strength density region corresponding to sign(λ_2_) < 0 did not show up in their plot. Some of these bonding features indeed appeared in their isosurface plot, which provide evidence of the presence of I···D(–C), I···C and I···N interactions (see Figs 1 and 2 of that study). They are also evident in the sign(λ_2_) × *ρ vs*. RDG plot of the study by Lee and coworkers^24^ (see the region −0.015 au < sign(λ_2_) × *ρ vs* <  +0.015 au of Fig. 4a of that study). However, for some reasons that are unclear, they provided no explanation of the underlying reasoning that caused the appearance of these isosurfaces and spikes.

From knowledge of the charge density in the bonding regions, the stability of the stable interactions follows the order Pb—I > I···D(–N) > I···D(–C) ≈ I···N(–C) > I···I > I···C(–N) in o-CD_3_ND_3_PbI_3_. This ordering is somewhat different to that found for o-CH_3_NH_3_PbI_3_ (Pb—I > I···H(–N) > I···H(–C) >  I···I > I···N(–C) > I···C(–N)). This is perhaps expected given the local intermolecular geometries of the two systems are somewhat different, an effect of temperature, which affect the distribution of charge density in the bonding regions. The stability orders noted above are in agreement with that of the delocalization indices calculated for these interactions (Fig. 5).

The intermolecular interactions predicted by DORI are compatible with QTAIM and RDG NCIs; hence the bond path topologies revealed using QTAIM are reliable indicators of the nature and types of noncovalent interactions involved in these systems.

While the results of QTAIM, RDG and DORI provide unequivocal evidence of the presence and nature of the noncovalent interactions, our results are markedly different from those reported by others^24,25,50^. The reason for this mismatch is certainly due to the widespread assumption that the ammonium H/D atoms are only atomic domains capable of forming significant noncovalent interactions.

### Natural bond orbital and charge transfer analysis

The Natural Bond Orbital (NBO) approach is another useful way of determining the nature and extent of donor-acceptor interactions^174,175^. These interactions emerge from the donation of electron density from the localized NBOs of the idealized Lewis structure into empty non-Lewis orbitals (and thus, to departures from the idealized Lewis structure description) and are referred to as “delocalization” corrections to the zeroth-order natural Lewis structure.

The stabilization energy *E*^(*2*)^ associated with delocalization between the lone-pair electron donating orbitals of the coordinated I atoms LP(I) and the anti-bonding σ*(C–D) fragments of d_6_-MA in o-CD_3_ND_3_PbI_3_ was found to vary between 0.50–5.50 kcal mol^−1^ for the I···D(–N) interactions. For the I···D(–C) interactions, the *E*^(*2*)^ for charge transfer delocalization between the lone-pair electron donating orbitals of Pb-coordinated I atoms and the σ*(C–D) orbital of d_6_-MA varies between 0.13 and 1.1 kcal mol^−1^. Similarly, for the I···N(–C) and I···C(–N) interactions, charge transfer delocalization is described by partial donation of lone-pair electron density from LP(I) to the anti-bonding σ*(C–N) fragment of the organic cation, with *E*^(*2*)^ = 0.13–0.15 kcal mol^−1^. In addition to these, our analysis shows that there is a delocalization between the bonding orbitals of Pb‒I and the anti-bonding σ* orbitals of the N–D and C–D bonds in d_6_-MA. The *E*^(*2*)^ for the σ(Pb‒I) → σ*(N–D) delocalizations range between 0.70 and 0.90 kcal mol^−1^, while those for the σ(Pb‒I) → *(C–D) delocalizations are between 0.12 and 0.83 kcal mol^−1^. Very similar results were obtained for o-CH_3_NH_3_PbI_3_.

Thus, the NBO second order analysis has recognized all the intermolecular interactions in o-CD_3_ND_3_PbI_3_ and o-CH_3_NH_3_PbI_3_ that were characterized with QTAIM and other charge density based approaches. The *E*^(*2*)^ values for O···H(–C) hydrogen bonds have been reported between 0.17 and 0.52 kcal mol^−1^ in non-perovskite systems that have binding energies ranging between −0.33 and −1.22 kcal mol^−1^^176^, demonstrating that the low polarity bonds can hydrogen bond with an electron rich site. This result allows us to conclude that the unusually large binding energies calculated for the CD_3_ND_3_PbI_3_ molecular models (Fig. 3) may not be very unrealistic since hyperconjugation energies for the charge transfer delocalization between the organic and inorganic moieties for the o-CD_3_ND_3_PbI_3_/o-CH_3_NH_3_PbI_3_ polymorphs are large (*vide supra*).

The formation of the CD_3_ND_3_PbI_3_ and CH_3_NH_3_PbI_3_ systems is accompanied by a transfer of approximately 0.24 *e*^*−*^ from the cuboctahedral inorganic core to each organic moiety, as inferred from a QTAIM population analysis. This is in agreement with other results^6,19^, confirming that intermolecular charge transfer is an important aspect of the perovskite system.

### Energy of hydrogen bonds from QTAIM based energy density analysis

As discussed above, in the perovskite structures examined a cation has a long-range interaction with an anion, so it is expected that the stabilization energy due to Columbic interaction is large. A question that arises is whether this electrostatically dominant contribution is independent of the strength of the hydrogen bonds, even though such bonds are generally recognized as predominantly ionic. There seems to be no obvious and straightforward way to separate the pure electrostatic cation-anion interaction from the hydrogen bond stabilization in an ion-pair system, including perovskites.

A second question that is worth considering in whether dispersion plays a significant role in stabilizing intermolecular interactions in CH_3_NH_3_PbI_3_ and in similar compounds such as CH_3_NH_3_SnI_3_. We have previously provided an answer to this question^18^. Due to the limitation of basis set associated with the software used, and to provide further insight and in the context of the current study, we performed a coupled cluster calculation on the gas-phase optimized cluster model called of CH_3_NH_3_SnI_3_ (roughly cubic). This calculation gave CCSD/cc-pVTZ(N, C, H)/cc-pVTZ-PP(Sn, I) binding energies of −104.92 and −101.88 kcal mol^−1^ for Δ*E* and Δ*E*(BSSE), respectively. The Symmetry Adapted Perturbation Theory (SAPT)^177^ level energy decomposition analysis, an approach that has gained widespread popularity in computational chemistry community, gave a value of −103.22 kcal mol^−1^ for the interaction energy. The decomposed energies were −99.93, +34.72, −26.61 and −19.95 kcal mol^−1^ for electrostatics, exchange repulsion, induction and dispersion, respectively, at the SAPT2 + 3/TZP level of theory. The close agreement between the CCSD and SAPT2 + 3 results, even evaluated with two different basis sets, indicates that dispersion and induction contributions are very substantial compared to any other hydrogen bonded interactions that have been reported in the literature. Note that London dispersion, as well as polarization, which constitutes the attractive part of the van der Waals potential, has long been under-appreciated in molecular chemistry as an important element of structural stability, yet has been shown to have a profound effect in chemical reactivity and catalysis^178^. The neglect was due to the common notion that both polarization and dispersion interactions are weak. This, in fact, may only be so for a single pair of weakly interacting atoms. For larger structures, the overall dispersion contribution grows rapidly with the size of the structure and can amount to tens of kcal mol^−1^^178^. For the molecular blocks we studied, the large contribution due to dispersion is not unexpected given that there are several heavy, readily polarizable atoms involved in the ion-pair formation. Because of the large induction and dispersion contributions, it is clear that the intermolecular interaction in the CH_3_NH_3_SnI_3_ ion-pair system, as well as that in CH_3_NH_3_PbI_3_, will contain a non-negligible amount of covalency. This is reasonable given a simple (H_2_O)_2_ dimer that has an experimentally determined binding energy of −5.0 kcal mol^−1^ bears partial covalency in the hydrogen bond that holds the two H_2_O molecules together^179^.

The relationship, *E*_b_ ≈ (1/2)*V*_b_, as proposed by Espinosa *et al*.^180^ has been used to calculate the strength of various intermolecular interactions in diverse chemical systems, where *V*_b_ is the potential energy density at the bond critical point. This relationship was used to calculate the strength of the noncovalent interactions responsible for the geometry of o-CD_3_ND_3_PbI_3_. For each of the two equivalent and the one shorter I···D(–N) bonds, interaction energies of −5.65 and −6.46 kcal mol^−1^, respectively, were calculated, so that the net interaction energy between the D atoms of the ammonium fragment and cage iodides is −17.76 kcal mol^−1^. Each of the two I···N(–C) pnictogen contacts has an interaction energy of −1.63 kcal mol^−1^, so there is a net contribution of −3.26 kcal mol^−1^ from these contacts to the structural stability. Similarly, for the two equivalent and one longer I···D(–C) contacts the interaction energies are −2.26 and −2.20 kcal mol^−1^, respectively. The interaction energies of the remaining two I···D(–C) contacts formed by the hydrogens of the methyl group in MA are calculated to be −1.88 kcal mol^−1^, each with an interaction energy of −0.94 kcal mol^−1^ and delocalization index of 0.021. Thus the net interaction energy between the methyl fragment and cage iodides is −8.60 kcal mol^−1^. Similarly, each of the two I···C(–N) pnictogen bonds has an interaction energy of −1.12 kcal mol^−1^, or a net contribution of −2.26 kcal mol^−1^ from these contacts to the overall stabilization energy. Moreover, each of the two I···I contacts contributes −0.56 kcal mol^−1^. Similar results were obtained for the corresponding interaction in o-CH_3_NH_3_PbI_3_. The energies estimated for the I···D(–N) bonds are far smaller than their corresponding hyperconjugative energies discussed above, suggesting that the interaction energies evaluated with *E*_b_ ≈ (1/2)*V*_b_ are not quantitatively realistic. They should not, however, be disregarded as they may convey important chemical information (at least qualitatively), as shown by others for assessing the strength of the hydrogen bonding interactions in the geometry of the tetragonal phase of CH_3_NH_3_PbI_3_^39^.

### Signatures from NMR analysis

The sign and magnitude of the two-bond spin-spin NMR scalar coupling constants *J* have been used to examine the presence of an intermolecular hydrogen bonding interaction^116,175,181–185^. These (^2h^*J*_I···N_ and ^2h^*J*_I···C_) were also examined to confirm the presence of both I···H(–C) and I···H(–N) hydrogen bonds formed by the methyl and ammonium groups of MA in o-CH_3_NH_3_PbI_3_. At the PBE/6-311 G**(N, H/D, C)/DZP(Pb, I) level of theory, ^1h^*J*_I···D(N)_ for the two equivalent and one shorter I···D(–N) contacts were calculated to be −6.1 and −8.8 Hz, respectively, whereas ^1h^*J*_I···D(C)_ for the two equivalent and one longer I···D(–C) contacts were found to be 3.1 and 2.6 Hz, respectively. Similarly, the indirect two-bond spin-spin coupling constant ^2h^*J*_I···N_ for the former interactions are 2.5 and 2.7 Hz, respectively, whereas the ^2h^*J*_I···C_ for the latter interactions are 0.8 and 3.1 Hz, respectively.

The NMR isotropic chemical shifts δ_iso_ is another important property often examined to see the nature of proton shielding or deshielding effect^186–188^. For the protons associated with the –NH_3_ and –CH_3_ groups in MA, the shifts were calculated at the same level of theory as *J*. For ^1^H spectra, a σ_ref_ value of 31.8 ppm was used, and the chemical shifts were estimated using the relationship δ_iso_ = σ_iso_ − σ_ref_. The ^1^H chemical shifts were calculated to be 7.8 and 8.8 ppm for the two equivalent and one shorter I···H(–N) contacts, respectively. The shifts for the two equivalent and one longer I···H(–C) contacts were 5.0 and 4.8 ppm, respectively. The high temperature MAS NMR chemical shifts associated with H atoms of –CH_3_ and –NH_3_ were reported to be 3.2(1) and 6.3(1) ppm, respectively^189^; these values were reported to be 3.4 and 6.4 ppm by Senocrate *et al*.^190^ and 3.5 and 6.5 ppm by Roiland *et al*.^72^, respectively. Although the calculated values of δ compared to the corresponding experimental ones are overestimated, which is not unusual^191^, yet there is a qualitative agreement between them. Similar results were obtained for o-CD_3_ND_3_PbI_3_. These results provide further evidence of the reliability of the assigned I···H/D(–C) and I···H/D(–N) intermolecular hydrogen bonding interactions in the perovskite compounds examined. That both the –CH_3_ and –NH_3_ groups interact with the iodides of the inorganic cage is supported by the NMR study of Senocrate and coworkers^190^, even though this conclusion was drawn for the system in a high temperature phase. The infrared spectroscopic study of Schuck *et al*.^192^ is in line with this finding; they suggested that the I···H–C hydrogen bond is relatively weaker than the I···H–N hydrogen bond in o-CH_3_NH_3_PbI_3_.

### Tilting of the BY_6_ octahedra and their relationship with hydrogen bonding

We now return to the question of tilting of the BY_6_^−4^ octahedra and whether hydrogen bonding interactions between the organic and inorganic moieties in o-CH_3_NH_3_PbI_3_, as well as those in other members of the BMY_3_ hybrid organic-inorganic perovskite family, are solely responsible for the *a*^*−*^*b*^+^*a*^*−*^ tilting pattern of the octahedra. This has already been partially answered by others^24,25^ in that the three Y···H(–N) hydrogen bonding interactions do explain the tilting pattern of the BY_6_^−4^ octahedra. According to Lee *et al*.^24^, because two of the three H atoms of the –NH_3_ fragment in MA form equivalent I···H(–N) bonds while the other forms a similar shorter bond with the perovskite iodides, this causes a rotation of the PbI_6_^4−^ octahedra in o-CH_3_NH_3_PbI_3_. Thus, the pattern of bonding between I_A_ and H_N_(1) atoms (referred to as mode 1 by Lee *et al*.^24^) and that between I_E_ and H_N_(2)/H_N_(2′) atoms (referred to as mode 2) corresponds with the pattern of iodide displacements; it was then suggested that the nature of the three hydrogen bonds correlated perfectly with the pattern of tilting observed for the orthorhombic geometry of the system, in which mode 1 induces only the anti-phase octahedral rotation while mode 2 correlates with both in-phase and anti-phase rotations.

While the existence of the three I···H(–N) bonds is unquestioned, perhaps an undue emphasis has been placed on their significance in order to match the predictions from theoretical calculations with the experimentally suggested observations^25,36^. The following is an example of the rationalization that has been advanced. “These three H···I bonds per MA ion are controlled by both the particular inorganic *a*^*−*^*b*^+^*a*^*−*^ tilt pattern and the organic MA conformation. Among these, the H_N_(1)···I_A_(1) hydrogen bond is the shortest (2.565 Å) and presumably the strongest. Accordingly, the N–H_N_(1)α···I_A_(1) angle is almost 180°. These results are in good agreement with the recent powder neutron diffraction study^31^, as well as the structure of ammonium iodide^193^ where hydrogen-bonding is known to play a key role in structural stabilization^194^. The N···I distances are much shorter than those of C···I and also the angle of C–H_C_(1)···I_A_(3) is smaller than that of N–H_N_(1)···I_A_(1). Accordingly, the hydrogen-bond interactions mainly originate from H atoms on nitrogen.” It was further suggested that the structure of MAPbI_3_ without the contribution from hydrogen bonding would remain *untilted* at all temperatures^24^. Therefore, patterns of hydrogen bonding can be used as an additional parameter to optimize photovoltaic and electronic properties in perovskites.

From our analyses presented in the previous subsections, however, we identified and characterized various other noncovalent interactions, in addition to the three I···H(–N) hydrogen bonds. Their occurrence is not unexpected given that the organic cation is entirely positive and electron deficient and has significant potential to accept electron density from the surrounding negative sites. Since the sizable cation is trapped inside an electron rich anionic iodide cage, yet does not cause the breaking of the Pb—I coordinate bonds (which happens with larger cations, leading to the development of materials in 2D^195^), it is not very surprising to see the development of other noncovalent interactions in the static orthorhombic geometry of the o-CH_3_NH_3_PbI_3_ system. Among these, the three I···H(–C) hydrogen bonds display a pattern comparable with that found for the three I···H(–N) hydrogen bonds (Fig. 2a). In this case, however, the two equivalent I···H(–C) bonds are slightly shorter than the other one. Apparently, and by analogy with the suggestion of Lee *et al*.^24^ quoted above, an immediate conclusion that comes to mind is that the three I···H(–C) hydrogen bonds could also be related to the *a*^*−*^*b*^+^*a*^*−*^ tilt pattern of the PbI_6_^−4^ octahedra. That is, the two equivalent and longer I···H(–C) bonds could be qualitatively correlated with the two types of octahedral rotations noted above. However, these sorts of correlations are superficial and at best purely qualitative. It actually makes little sense to directly connect any one type of hydrogen bond pattern with the tilting pattern of the PbI_6_^4−^ octahedra and then at the same, neglect all other remaining interactions.

It should be kept in mind that the inorganic *a*^*−*^*b*^+^*a*^*−*^ tilt pattern is a low-temperature feature present in many inorganic halide and oxide perovskites. The tilting of the octahedra these systems occurs in 23 different ways following the nomenclature introduced by Glazer^33^, which in turn leads to 15 different space groups. They result from symmetrically distinct combinations of the out-of-phase and in-phase tilts (rotations) of the corner-sharing octahedra along the three crystallographic directions, and are caused by symmetry breaking due to R_4_^+^ and M_3_^+^ normal displacement modes associated with these tilts, respectively. The details of the tilting nomenclature of perovskites based on crystal structure and composition have recently been discussed by Mitchell and coworkers^196^. The *a*^0^*a*^0^*a*^0^ (*Pm* $\bar{3}\,$*m* space group) pattern is the ideal untilted structure with cubic symmetry. Of the 15 potential tilts, the tilting patterns *a*^0^*b*^+^*b*^+^, *a*^*−*^*a*^*−*^*c*^*−*^ and *a*^+^*b*^+^*b*^*−*^ in Glazer notation, have not been observed in bulk materials. (The symbols + and – represent the in- and anti-phase tilting of the metal centered octahedra, respectively.) Also, the *a*^0^*b*^+^*b*^*−*^ pattern has never been experimentally observed and perhaps should not appear in a list of possible tilt systems of the perovskite structure. While there are 59 orthorhombic space groups, a great number of experimentally determined orthorhombic perovskites belong to, among others, *Pbnm* (viz. CaGeO_3_^197^, DyFeO_3_, HoFeO_3_^198^, M′VO_3_ (M′ = Y, Nd, Tb)^199^, CaTiO_3_)^200^ and *Pnma* (viz. LaMO_3_ (M = Ho, Er, Tm, Yb, Lu)^201^, CaTiO_3_^202^, YAlO_3_, RFeO_3_ (R = La, Pr, Nd, Sm, Eu, Y)^201^, in which the perovskite geometries belonging to these two space groups have exactly the same symmetry, but with the crystallographic axes defined differently. Systems with *Pnma* space group display tilt patterns such as *a*^*−*^*a*^*−*^*c*^+^ and *a*^*−*^*b*^+^*a*^*−*^, all with octahedral rotations in their ground states. According to Woodward^203^, the *a*^*−*^*a*^*−*^*c*^+^ tilt pattern maximizes B–O covalent bonding interactions and minimizes B–O repulsion in BMO_3_ perovskites. The B-site displacements in *Pnma* play an important role in minimizing M–O repulsion and optimizing the M-site coordination environment, explaining why do so many perovskites adopt the *Pnma* structure^204^.

Computationally, o-CH_3_NH_3_PbI_3_ has been suggested to be the most stable phase compared to all other temperature-dependent phases of MAPbI_3_, and that this result is in agreement with experimental observations. For example, halide perovskites generally crystallized in three different phases, known as the γ, β and α phases, and associated with the high, intermediate and low temperature phases, respectively. For MAPbI_3_, these correspond to the orthorhombic, tetragonal and cubic phases, which are stable in the temperature ranges *T* < 163 K, 163 < *T* < 327 K and *T* > 327 K, respectively^32,205^. Similarly, for the CsSnI_3_ all-inorganic perovskite, such temperature-dependent phases have been known experimentally with transition temperatures of 352 (*Pnma*, orthorhombic), 431 (*P4/mbm*, tetragonal) and 440 K (*Pm* $\bar{3}\,$*m*, cubic), respectively^206^. The structures of both α and β phases of CsMX_3_ (M = Sn, Pb; X = Cl, Br, I) perovskites are dynamically unstable, as observed for MAPbI_3_, whereas the geometry of the γ phase is stable.

The *a*^*−*^*b*^+^*a*^*−*^ tilt pattern shows up regardless of the nature of the B-site cation in the perovskite geometries. It also occurs both in CsMX_3_ (M = Sn, Pb) and in MAPbY_3_. The net rotation of the MY_6_^4−^ octahedra in perovskites cannot just be due either to lattice dynamics, or to intermolecular/coordinate bonding interactions. It is rather the consequence of the effect of both, largely due to the former with an appreciable effect due to the latter. There is precedence that this kind of tilting of the metal-coordinated octahedra appears in systems even without hydrogen and B-site coordinate bonding and without a B-site cation (*vide infra*). In other words, one can view this as a local feature and the extent of this is controlled by several factors. These include, *inter alia*, the extent of covalent and ionic bonding between the metal cation and the coordinating anions forming the inorganic perovskite cage, the M and B site strains and their sizes, the nature of the organic or inorganic cation, the displacement of the B site cation from the center of the inorganic cage, and the nature of the intermolecular interaction involved (such as dative coordinate bonding due to inorganic cations or hydrogen bonding due to organic or inorganic cations provided the B-site species contains H atoms). Many have traditionally attributed the origin of tilting to a steric origin (for example^207^), while others suggested it is a consequence of a mismatch of ionic radii^204,208^.

In support of this, we note that Cammarata and Rondinelli^209^ have correlated the covalency of the metal–oxygen bond in some *Pbnm* ferrates with the octahedral rotation amplitudes and found that the larger the covalency component in the Fe–O bond, the less distorted the structure; this would be associated with relatively stronger long-range inter-octahedral (Fe–O–Fe) interactions. Similarly, for Pb-based compounds, it was shown that the greater the extent of volume contraction passing from the high temperature phase to the low-temperature phase, the greater the octahedral tilting since it is associated with a higher degree of iconicity^210^. This attribute has assisted in providing some insight into the reason why Pb–I–Pb angles in Pb-based perovskites are more tilted compared to that of the Sn–I–Sn angles in Sn-based perovskites^21,28^. This bonding feature is in contrast with rationalizations for the oxide-based perovskites, where the degree of ionicity was correlated with the degree of octahedral tilting, and an increase in the degree of covalency was correlated with the linearity of the corner sharing O–M(–O) bonds^28^. Because tilting breaks ideal σ-bonding between the metal and its coordinate bonding partners, it facilitates the development of orbital type interactions^207,211^, and the idea that linear bonds are more covalent than non-linear bonds is not a well developed concept and that no robust experimental and theoretical approaches have so far been developed to accurately quantify ionicity and covalency in metal-ligand interactions in metal coordinated systems, as well as in noncovalent interactions. What has been done so far is tentative, and is nothing other than a researcher biased interpretation. Explanations concerning the ionic and covalent nature of chemical interactions have, of course, been advanced based on molecular orbital pictures; some have been based on electron density analysis; some on electron delocalization indices; and some on other approaches. Sometimes the results obtained from one approach do not match with the results from other approaches, leading to some controversy. Although most of these approaches have helped to explain to some extent the experimentally observed trends in several systems, they have produced questionable results for other systems.

The origin of octahedral tilting has been discussed many times in the case of orthorhombic BMO_3_ perovskites. According to Abakumov and coworkers^212^, a primary reason for the tilting distortion is the mismatch between the B−O and M−O interatomic distances in BMO_3_ perovskites, which can be inferred from the tolerance factor. Many halide and oxide perovskites with widely varying composition exhibit this kind of ground-state tilting pattern, especially when their Goldschmidt tolerance factors *τ* are <1; this is not very different to what has been observed for CH_3_NH_3_PbI_3_. The tolerance factor τ is calculated by $\tau =\frac{1}{\sqrt{2}}\frac{{R}_{B}+{R}_{Y}}{{R}_{M}+{R}_{Y}}$, where *R*_*B*_, *R*_*M*_, and *R*_*Y*_ are ionic radii of B, M and Y site ions, respectively. For ideal cubic structures, τ = 1. However, in the case of structures suffering from tilting and ferroelectric distortions, *τ* < 1 and >1, respectively. While this condition is satisfied for many systems, there are cases where it fails to provide the expected result. For instance, *τ* is >1 for SrTiO_3_, SrRuO_3_, CaMnO_3_, and LaMO_3_ (M = Al, Cu, Co, Ni), but tilting is evident of all these systems. The range 0.8 ≤ *τ* ≤ 1 usually applies to perovskites, although the lower part of this range is generally found to be associated with lower symmetry tilted structures^213^.

There are three types of geometrical distortions that have been discussed in the literature: second-order Jahn-Teller distortion, ferroelectric distortion, and tilting. The first two are electronic in nature while the origin of the latter is steric^214^. Such tilting dramatically improves B-site coordination, and possibly improves electrostatic binding by facilitating significant volume contraction. While the ground-state tilting pattern in perovskites is widespread, does this mean that I···H–N interactions cause the octahedral tilting and that without them there would be no tilt at all? Clearly, this is not the case; the system would display tilting as long as the space group symmetry is constrained to orthorhombic.

**Figure 11:**
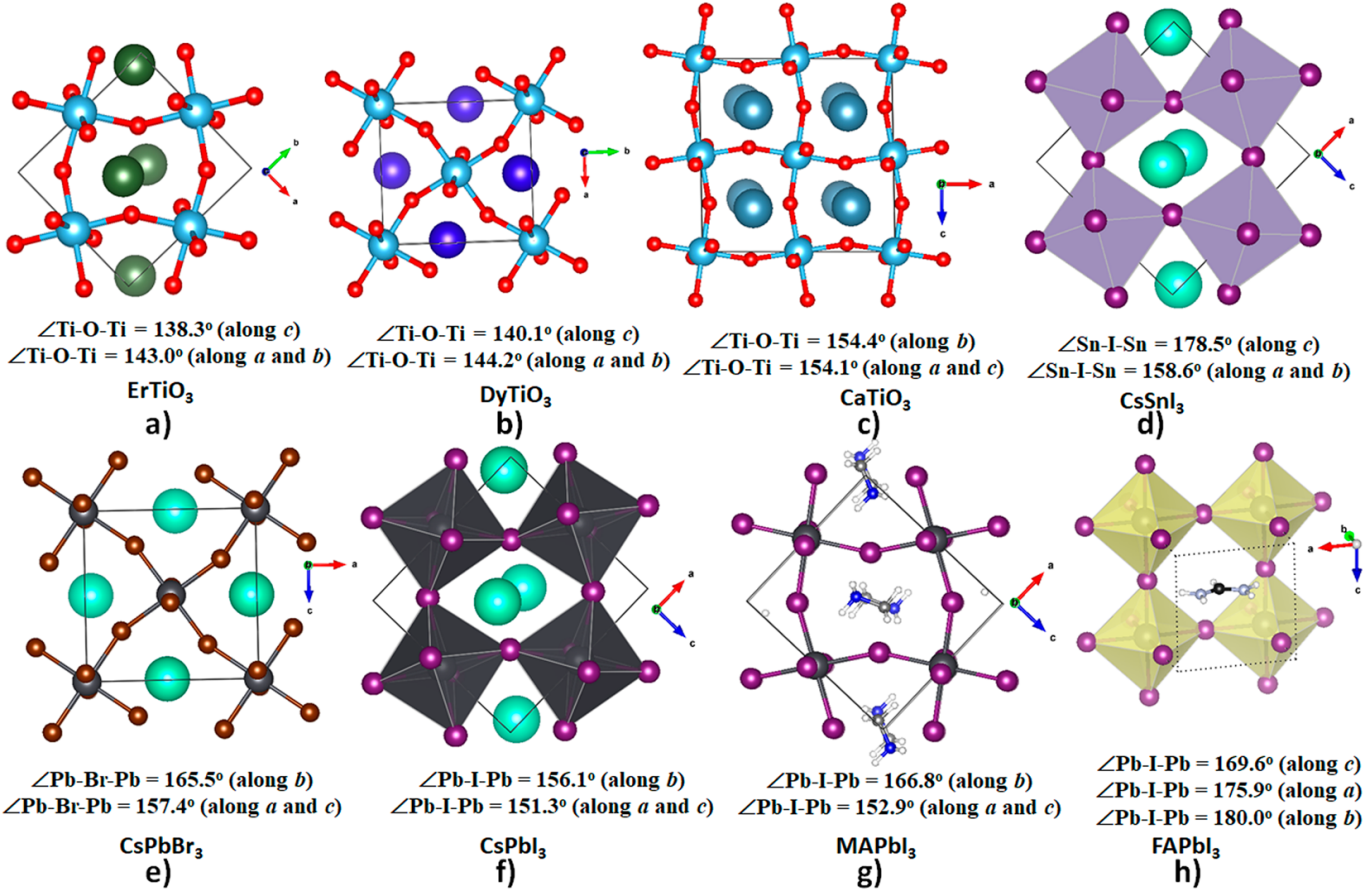
The computed structures of (**a**–**d**) were obtained from the Materials Project Database^218^ with ref. IDs mp-756296 (10.17188/1290469), mp-779599 (10.17188/1268423), mp-4019 (10.17188/1207766) and mp-568570 (10.17188/1274524), respectively. The experimental structure of (**e**) is taken from ref.^240^, whereas the computed structures of (**f**–**g** and **h**) were emerged from this study using PBE (10 × 8 × 10 *k*-point mesh) and from ref.^241^, respectively. The tilting angle (in degree) is shown in each case.

To clarify this further, consider Fig. 11. It presents both inorganic halide and oxide and organic-inorganic halide perovskites in the orthorhombic phase, including the eponymous mineral perovskite CaTiO_3_. The latter adopts an orthorhombic distorted-perovskite structure with space group *Pbnm*, a structure that is common in perovskite oxides. It shows the combination of two kinds of TiO_6_ octahedron tilts, two out-of-phase and one in-phase, with the *a*^*−*^*a*^*−*^*c*^+^ pattern^215^. While the first five perovskite systems of Fig. 11 do not contain an organic cation, these all have tilted octahedra (either BO_6_ or BY_6_), the origin of which is known to be primarily due to lattice disorder. However, the effect of coordination bonding between the inorganic cation and the cage oxides or halides is not insignificant as it is involved with a many-fold bonding topology that influences the extent of tilting and the volume of the lattice. Coordination of the B-site cation reported using bond valence analysis of the first coordination sphere can range from 8 to 12. Many naturally-occurring BMO_3_ and BMX_3_ perovskite-group minerals, including CaTiO_3_, that adopt the orthorhombic structure display the B-site cations involved in 8-fold coordination^216,217^. SrTiO_3_ perovskite can be described as consisting of corner sharing MO_6_ octahedra with the B cation occupying the 12-fold coordination site formed in the middle of the cube of eight such octahedra.

The magnitude of the bond angles shown in Fig. 11 suggests that, depending on the nature of packing and size of the atomic domains, tilting of octahedra can be either severe in one direction or less pronounced in the other, driven by the nature of coordination between the entrapped guest and the host. This is evident in the CaTiO_3_, CsPbI_3_, CsSnI_3_ and CsPbBr_3_ systems that have an inorganic cation, yet the extent of tilting is not the same since the sizes of the guest cation, the metal ion and the ligators all compete with each other to determine the extent to which a given system tilts and where the B-site cation is positioned so as to maximize the bonding interaction between the interacting constituents in the equilibrium geometry of the system. A similar argument can be made for octahedral tilting in o-CH_3_NH_3_PbI_3_ and o-CH_3_NH_3_SnI_3_. Consequently, the origin of the tilting thus cannot be *solely* attributed to I···H–N hydrogen bonding, or *solely* to any other type of noncovalent interaction identified.

**Figure 12:**
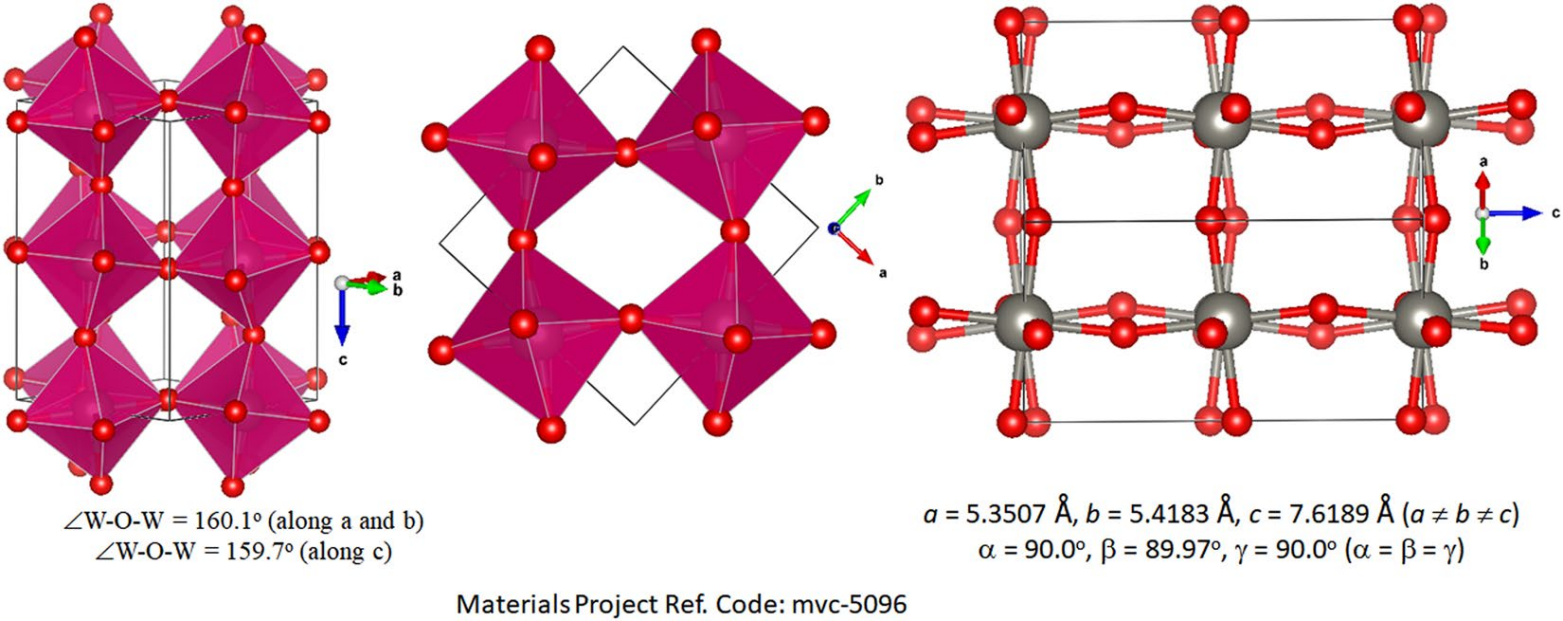
The orthorhombic (*Pmna*) geometry of WO_3_, showing a perovskite-like architecture without the presence of any B-site cation inside the cage. The tilting angles and lattice constants are shown.

Now let us consider the widely known system, WO_3_. This is a semiconducting material, and has a bandgap of 1.676 eV, and a formation energy of −2.179 eV atom^‒1^, evaluated theoretically with GGA + U^218^. Figure 12 illustrates the theoretically modeled *Pnma* structure of the system (16 atoms in the unit-cell). Several phases of this system were experimentally characterized and analysed by exploring the sequence of temperature-induced phase transitions in WO_3_^219^. They were rationalized in terms of changes in the octahedral tilt of the system and/or displacements of W from the center of the WO_6_ octahedron. As such, *β*-WO_3_ (*Pbcn*) was observed at 350 °C, with an *a*^0^*b*^+^*c*^−^ tilt. The tetragonal *α*-WO_3_ (*P*4/*ncc*) was observed at 800 °C, with an *a*^0^*a*^0^*c*^−^ tilt^220^. Similarly, the low-temperature ε and δ phases were identified to be monoclinic and triclinic and both are associated with an *a*^−^*b*^−^*c*^−^ tilt. As can be seen from Fig. 12, the orthorhombic geometry of this system is perovskite-like, with a tilt of the WO_6_ octahedra similar to that in o-CsSnI_3_ and BTiO_3_ (B = Er, Dy). The ∠W-O-W is 160.1° along the *a* and *b* axes, and is 159.7° along the *c* axis, and all deviate significantly from 180° as a result of tilting. This structure of WO_3_ is an example of a structure that does not contain any organic or inorganic cation in its cage interior, and so suffers from a deficiency in the B-site population; analogous systems of this type include ReO_3_^218,221,222^ and ScF_3_^223^. The absence of a guest species in these systems is not unusual since the metal cores in these have a formal oxidation state of +6. Quite evidently, WO_3_ does not experience the effects of any hydrogen bonding or coordinate bonding between the O and B site species, which would be expected when the W–O cage accommodates a B-site species. While we did not explore the impact of hydrogen or coordinate bonding in this system, which could be done if a neutral organic or inorganic species is doped inside the cage, it is reasonable to conclude that tilting of the octahedra of WO_3_ is the consequence of lattice disorder caused by the delicate balance between extent of attractive and repulsive interactions between the interacting sites at the atomic level. One cannot simply fully attribute this to what has traditionally been called a steric effect. This is because this effect cannot explain tilting on a system by system basis. We note further that the metal halide octahedra in perovskites in one dimension can be corner-sharing, edge-sharing, or face-sharing to form a 1D nanowire surrounded by organic cations. Their configurations can be either linear or zigzag, and their chemical formulae are variable depending on the connecting methods and the organic cations chosen^224^. Although many recent examples of this are available^225–227^, we note in particular that CsPbI_3_ adopts a distorted nanowire-like structure in one dimension that unambiguously does not involve H-bonding^228^.

Figure 11h illustrates the structure of formamidinium halide perovskite, CH(NH_2_)_2_PbI_3_ (also known as FAPbI_3_), which may be used as another case to determine whether hydrogen bonding is the cause of octahedral tilting. The B-site molecular cation FA in this system is larger than MA (tentative ionic radii of MA and FA are 2.16 and 2.53 Å, respectively)^229,230^. As the H atoms of MA make noncovalent interactions with the iodides in o-MAPbI_3_, the H atoms on FA form hydrogen bonding interactions with the iodides of the inorganic iodide framework. While some are of the view that the diamine may hydrogen bond to the halogen atoms to both sides of the cage^231^, others have argued these are indeed present but probably weaker than those in MAPbI_3_^53^. This system indeed shows octahedral tilting, yet Lee *et al*.^24^ have attributed this to the engineered direction and strength of hydrogen bonds that disfavors significant octahedral tilting in this system, since these are formed on both sides of the molecule^232^, rendering the system pseudocubic. Nevertheless, multiple experimental results reveal that the ground state of the system involves complex disorder at low temperature. Because the symmetry of the FA cation is geometrically frustrated, this causes the system to adopt *P4/mbm* space group symmetry that lacks the *a*^*−*^*b*^+^*a*^*−*^ tilting pattern^53^. This is indeed in contrast to MAPbI_3_/MASnI_3_ where the molecular symmetry and its size are compatible with tilting, showing the importance and role played by the molecular cations in a different hybrid organic-inorganic perovskite system. The replacement of MA by FA results in a global “locking” of the PbI_6_ octahedra tilting in the resulting perovskite, restricting the rotational or tumbling motion of the CH(NH_2_)_2_^+^ molecular ion in a locked cage^233^ due to the lattice dynamics that are significantly reduced. In the mixed B-site cation perovskites, there is also an increase in I···H−N hydrogen bonding interactions between FA and the Pb−I/Sn−I lattice, in which, it is suggested^234–236^ confering greater structural stability.

While there are suggestions that the average crystal structure of FAPbI_3_ is cubic^217^, this cannot be so since the lattice constants are unequal, signifying local octahedra tilts expected of an orthorhombic structure^94,237^. The simulation results of Ghosh and coworkers^238^ are also in agreement with this since FAPbI_3_ adopts a tilted structure with an average tilt angle of 11°, which is very similar to the results from another study^237^. In this^237^, as well as in another report^238^, it is predicted that when cations such as Cs^+^ and Rb^+^ are introduced into the B-sites they are less able to fill the space in the cage, and as a result the octahedra tilts further to pack more effectively, causing the tilt angle to increase from 11° in FAPbI_3_ to 20° in FA_0.9_Rb_0.1_PbI_3_.

**Figure 13:**
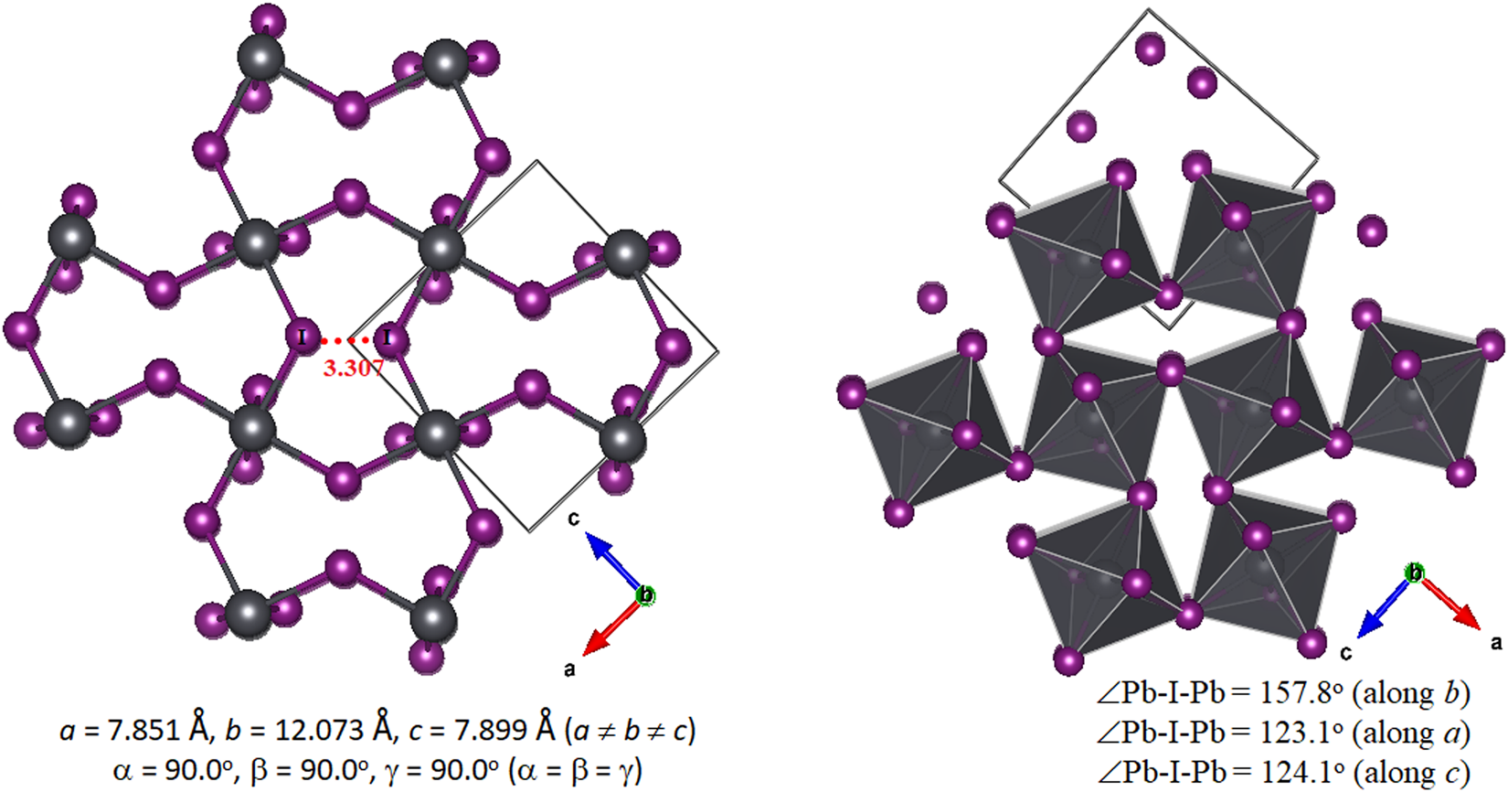
Two views of the PBE relaxed geometry of o-CH_3_NH_3_PbI_3_ without the MA cation, showing a significantly distorted orthorhombic structure. The lattice constants, the shortest I···I distance (in Å) (left) and tilting angles along the three crystallographic axes (right) are shown.

In order to provide more insight into the whether the absence of the organic cation can cause o-CH_3_NH_3_PbI_3_ to become *untilted*, we removed the organic cation from the o-CH_3_NH_3_PbI_3_ geometry, and relaxed it at the same PBE level of theory as was done for the system with the MA cation. The resulting hypothetical structure is illustrated in Fig. 13. The removal of the B-site cation has significantly decreased the volume of the system to 748.75 Å^3^, compared to a value of 1026.46 Å^3^ computed for o-CH_3_NH_3_PbI_3_. The decrease in volume did not change the orthorhombic nature of the resulting geometry. Interestingly, the decrease in the cell volume results in an increase in the bandgap to 2.1 eV. While the nature of the bandgap is predicted to be indirect, it is a result of the shift of the position of CBM. For instance, the bottom of the conduction band occurs at the *k*-point of (1/2, 0, 1/2) in reciprocal coordinates, whereas the top of the valence band is a minority spin state with energy and occurs at the *k*-point of (0, 0, 0). This is indeed markedly different to the PBE bandgap value of 1.78 eV computed for o-CH_3_NH_3_PbI_3_, which is direct at high symmetry *Γ*-point in *k*-space.

It is readily seen from Fig. 13 that the PbI_6_^4−^ octahedra in the perovskite-like structure are severely tilted due to the removal of the organic cation. Although the ∠Pb–I–Pb tilting angles along the *a* and *c* axes are not exactly the same, they are nearly equivalent. They are both markedly different from the tilting angle found along the *b* axis. Moreover, the calculated I···I distances (marked in red) are 3.307 Å, much less than twice the van der Waals radius the I atom, 4.08 Å, suggesting that these I atoms are bonded to each other, and that this is responsible in part for the severity of tilting of the PbI_6_^4−^ octahedra (in the B-site free perovskite). o-CH_3_NH_3_PbI_3_, on the other hand, shows an analogous tilting of the same octahedra with MA, yet the I···I interaction distances are larger, and is evidently caused by the organic cation by pushing the I atoms apart and for its accommodation within the cage. Nevertheless, both tilting and I···I contacts are an inherent feature of the system; consequently, the importance of the latter motif should not be neglected. These results, contrary to those of Lee *et al*.^24^, suggest that the absence of the organic cation would result in a *tilted* system.

The discussion given above leads to the conclusion that tilting of the MY_6_ octahedra in hybrid organic-inorganic BMY_3_ perovskites is not entirely caused either by intermolecular hydrogen bonding, or by dative coordinate bonding interactions. This does not mean these do not play a role in driving the extent of tilting in halide perovskites. The chemical bonding of a variable-sized B-site cation actually cooperates with lattice dynamics to favor distortion, thus contributing to tilting and to the properties of the resulting systems at equilibrium. The study by Shojaei *et al*.^239^ might provide additional insight into the chemical physics and physical chemistry of octahedral tilting in perovskites, and the way it is correlated with other properties of these systems.

One of the major problems with the current state of the art of theoretical and experimental methods is that none is sufficiently robust to provide accurate information on the exact nature of lattice dynamics^74^, as well the dynamic nature of hydrogen bonds, which are collectively responsible for the extent of octahedral tilting that influences the functional properties of the system. The lattice and B-site cation dynamics, and how they determine tilting, have been the subject of many discussions, but these have not answered the question how and in what way exactly they tune the functional properties of the system. For instance, the study of Maughan and coworkers that used X-ray pair distribution function data was unable to unequivocally determine if the octahedral tilting disorder in iodide-based vacancy-ordered double perovskite compounds is static or dynamic^27^. Li and Rinke^37^ have recently demonstrated that the interplay between octahedron tilting and the MA dynamics still needs a clearer understanding, referring to this as an example of the chicken-and-egg paradox. Specifically, the study showed that the low symmetry of the organic cation (the chicken) triggers the inorganic-framework (the egg) deformation, whose magnitude is sensitive to the orientation of the organic cation; this deformation then aids the overall stabilization of the hybrid perovskite structure. The final location of the organic cation was assumed to be very sensitive to the inorganic-framework deformation, thereby leaving the authors unable to say which came first, the deformation of the inorganic-framework or position of the organic cation. To this end and from the results of our calculations provided above, it can be said with some certainty that the deformation of the lattice is an inherent feature of the o-MAPbI_3_ system; this suggests that this comes first. The second part is the MA cation, whose molecular size and symmetry determine the way it is to be accommodated inside the perovskite cage to maintain an appropriate size-match selectivity, and the extent to which it would influence octahedral tilting and determine the properties of the system.

## Conclusion

Several descriptors of bonding interactions that are currently in use for the study of a variety of noncovalent interactions, including those recommended by IUPAC, were invoked to reveal them in o-CH_3_NH_3_PbI_3_ and its deuterated analogue o-CD_3_ND_3_PbI_3_, as well as to validate the usefulness of the current state-of-the-art computational tools adopted. We demonstrated that the I···H(–C)/I···D(–C) interactions in the static geometry of the perovskite polymorph o-CH_3_NH_3_PbI_3_/o-CD_3_ND_3_PbI_3_ are not insignificant as is often assumed. The presence of I···H(–N) and I···D(–N) interactions in the experimental neutron diffraction geometries of o-CH_3_NH_3_PbI_3_^31^ and its fully deuterated analogue was probably assigned based on the “less than the sum of the van der Waals radii” concept and several other weak interactions were overlooked; this is misleading for various reasons described in this paper. It is therefore recommended that this criterion *alone* should not be used to analyze chemical bonding in systems such as the one examined in this study. To this end, we showed that it is always instructive to perform several analyses using available computational procedures in order to arrive at definitive conclusions on the nature and type of noncovalent interactions being explored.

Given the geometrical complexity of the o-CH_3_NH_3_PbI_3_ system, it is not easy to accurately model or measure the (energetic) strength of various intermolecular interactions in this system, nor it is straightforward to determine how these interactions individually influence tilting since there are many of them that are collectively and simultaneously responsible for this effect. Based on the demonstrations widely available in the literature on noncovalent interactions^124,125,129–134^, it is evident that it is incorrect to assume that strong noncovalent interactions are always important and weak interactions can be neglected to explain a physical effect. However, from the energies obtained from standard molecular model calculations, it can be concluded with some confidence that the strong competition between the methyl and ammonium groups of the organic cation forming the I···H(–C)/I···D(–C) and I···H(–N)/I···D(–N) hydrogen/deuterium bonds, as well as the carbon- and pnictogen bonds, explain why the organic cation in o-CH_3_NH_3_PbI_3_/o-CD_3_ND_3_PbI_3_ is located near (or slightly displaced from) the centre of the perovskite cage at low temperatures. The position of the cation is not the same as that found in the geometries of the tetragonal and pseudo-cubic polymorphs of the corresponding system, since the effect of temperature drives its positioning, leading to the formation of different modes of intermolecular interactions between the host and guest species. We plan to explore this, and report it elsewhere.

We conclude that the various noncovalent interactions revealed in this study do not by themselves produce the tilting effect; rather their presence or absence respectively decreases or increases the extent of tilting. This was shown by removing the organic cation and by comparing the geometrical results of the resulting hypothetical system with those of the same system that has the cation in it. The removal of the cation significantly increased the bandgap of the resulting material and caused the nature of the bandgap to become indirect. This is consistent with many reports that suggest that the larger the tilting the larger the bandgap and hence the larger the carrier masses, thereby affecting the photovoltaic properties. While the tilting phenomenon is known to control photo-carrier electron-hole effective masses, band gap and other photovoltaic properties of halide perovskites^21–23^, it is certainly not controlled just by the I···H(–N) interactions. It should be appreciated that the tilting in o-CH_3_NH_3_PbI_3_/o-CD_3_ND_3_PbI_3_ triiodide perovskite is a complex phenomenon that is driven by the interplay between many interactions, including those that arise from lattice and B-site cation dynamics even at low temperatures^72^.
